# Supplementary material for: How can human-centered design build a story-based video intervention that addresses vaccine hesitancy and bolsters vaccine confidence in the Philippines? A mixed method protocol for project SALUBONG
Source: BMJ Open. 2021 Jun 8;11(6):e046814. doi: 10.1136/bmjopen-2020-046814 (PMC8190986; doi:10.1136/bmjopen-2020-046814)
Supplement: Author's manuscript [file bmjopen-2020-046814.draft_revisions.pdf]

BMJ Open is committed to open peer review. As part of this commitment we make the peer review history of every article we publish publicly available.

When an article is published we post the peer reviewers' comments and the authors' responses online. We also post the versions of the paper that were used during peer review. These are the versions that the peer review comments apply to.

The versions of the paper that follow are the versions that were submitted during the peer review process. They are not the versions of record or the final published versions. They should not be cited or distributed as the published version of this manuscript.

BMJ Open is an open access journal and the full, final, typeset and author-corrected version of record of the manuscript is available on our site with no access controls, subscription charges or pay-per-view fees (http://bmjopen.bmj.com).

If you have any questions on BMJ Open's open peer review process please email info.bmjopen@bmj.com

BMJ Open

Project SALUBONG: Using human-centered design to build vaccine confidence via empathy and narratives in the Philippines

Journal:	*BMJ Open*
Manuscript ID	bmjopen-2020-046814
Article Type:	Protocol
Date Submitted by the Author:	10-Nov-2020
Complete List of Authors:	Reñosa, Mark Donald; Research Institute for Tropical Medicine, Department of Epidemiology and Biostatistics; Ruprecht Karls Universitat Heidelberg, Institute of Global Health<br>Wachinger, Jonas; Ruprecht Karls Universitat Heidelberg, Institute of Global Health<br>Bärnighausen, Kate; Ruprecht Karls Universitat Heidelberg, Institute of Global Health; University of the Witwatersrand, School of Public Health<br>Aligato, Mila; Research Institute for Tropical Medicine, Department of Epidemiology and Biostatistics<br>Landicho-Guevarra, Jhoys; Research Institute for Tropical Medicine, Department of Epidemiology and Biostatistics<br>Endoma, Vivienne; Research Institute for Tropical Medicine, Department of Epidemiology and Biostatistics<br>Landicho, Jeniffer; Research Institute for Tropical Medicine, Department of Epidemiology and Biostatistics<br>Bravo, Thea Andrea; Research Institute for Tropical Medicine, Department of Epidemiology and Biostatistics<br>Demonteverde, Maria Paz; Research Institute for Tropical Medicine, Department of Epidemiology and Biostatistics<br>Guevarra, Jerric Rhazel; Research Institute for Tropical Medicine, Department of Epidemiology and Biostatistics<br>de Claro, Nicanor III; Research Institute for Tropical Medicine, Department of Epidemiology and Biostatistics<br>Inobaya, Marianette; Research Institute for Tropical Medicine, Department of Epidemiology and Biostatistics<br>Adam, Maya; Stanford University School of Medicine<br>Chase, Rachel; Johns Hopkins University Bloomberg School of Public Health<br>McMahon, Shannon; Ruprecht Karls Universitat Heidelberg, Institute of Global Health; Johns Hopkins University Bloomberg School of Public Health
Keywords:	Protocols & guidelines < HEALTH SERVICES ADMINISTRATION & MANAGEMENT, Public health < INFECTIOUS DISEASES, HEALTH SERVICES ADMINISTRATION & MANAGEMENT, Health policy < HEALTH SERVICES ADMINISTRATION & MANAGEMENT, Health & safety < HEALTH SERVICES ADMINISTRATION & MANAGEMENT, Organisation of health services < HEALTH SERVICES ADMINISTRATION & MANAGEMENT

I, the Submitting Author has the right to grant and does grant on behalf of all authors of the Work (as defined in the below author licence), an exclusive licence and/or a non-exclusive licence for contributions from authors who are: i) UK Crown employees; ii) where BMJ has agreed a CC-BY licence shall apply, and/or iii) in accordance with the terms applicable for US Federal Government officers or employees acting as part of their official duties; on a worldwide, perpetual, irrevocable, royalty-free basis to BMJ Publishing Group Ltd ("BMJ") its licensees and where the relevant Journal is co-owned by BMJ to the co-owners of the Journal, to publish the Work in this journal and any other BMJ products and to exploit all rights, as set out in our licence.

The Submitting Author accepts and understands that any supply made under these terms is made by BMJ to the Submitting Author unless you are acting as an employee on behalf of your employer or a postgraduate student of an affiliated institution which is paying any applicable article publishing charge ("APC") for Open Access articles. Where the Submitting Author wishes to make the Work available on an Open Access basis (and intends to pay the relevant APC), the terms of reuse of such Open Access shall be governed by a Creative Commons licence – details of these licences and which Creative Commons licence will apply to this Work are set out in our licence referred to above.

Other than as permitted in any relevant BMJ Author's Self Archiving Policies, I confirm this Work has not been accepted for publication elsewhere, is not being considered for publication elsewhere and does not duplicate material already published. I confirm all authors consent to publication of this Work and authorise the granting of this licence.

For peer review only

Title:

Project SALUBONG: Using human-centered design to build vaccine confidence via empathy and narratives in the Philippines

Name of Authors and Affiliations:

Mark Donald C. Reñosa[1,2], Jonas Wachinger[2], Kate Bärnighausen[2,3], Mila F. Aligato[1], Jhoys Landicho-Guevarra[1], Vivienne Endoma[1], Jeniffer Landicho[1], Thea Andrea Bravo[1], Maria Paz Demonteverde[1], Jerric Rhazel Guevarra[1], Nicanor de Claro III[1], Marianette T. Inobaya[1], Maya Adam[4], Rachel Chase[5], and Shannon A. McMahon[2,5]

[1]Department of Epidemiology and Biostatistics, Research Institute for Tropical Medicine – Department of Health, Muntinlupa, Philippines.

[2]Heidelberg Institute of Global Health, Ruprecht-Karls-Universität Heidelberg, Heidelberg, Germany.

[3]School of Public Health, University of the Witwatersrand, Johannesburg, South Africa

[4]Department of Pediatrics, Stanford University School of Medicine, Stanford, USA.

[5]International Health Department, Johns Hopkins Bloomberg School of Public Health, Baltimore, USA.

Corresponding Author:

Shannon A. McMahon, Ruprecht-Karls-Universität, Heidelberg Institute of Global Health, University Clinic Heidelberg, INF 130.3, D 69120 Heidelberg, mcmahon@uni-heidelberg.de; Phone: +49 (0)6221 56-35049.

For peer review only

ABSTRACT

Introduction: Since the onset of a dengue vaccine controversy in late 2017, vaccine confidence has plummeted in the Philippines, leading to a measles and polio outbreak in early 2019. This protocol outlines a human-centered design (HCD) approach to co-create and test an intervention that addresses vaccine hesitancy (VH) via narrative and empathy with and among families and health care workers.

Methods and Analysis: "Salubong" is a Filipino term that means to welcome someone back into one's life, reinforcing notions of family ties and friendships. We apply this sentiment to vaccines. Following the four phases of HCD, guided by a theoretical framework, and drawing from locally-held understandings of faith and acceptance, we will conduct in-depth interviews (IDIs) and focus group discussions (FGDs) in rural and urban Filipino communities that witnessed dramatic increases in measles cases in recent years. During qualitative engagements with patients, providers and policymakers, we will collect narratives about family and community perceptions of childhood vaccinations, public health systems, and opportunities to restore faith. IDIs and FGDs will continuously inform the development of (and the delivery mechanisms for) story-based interventions. Once developed, we will test our co-created intervention(s) among 600 families and administer a VH questionnaire prior to and immediately following the intervention encounter. We will use the feedback gained through the survey and Kano-style questionnaires to further refine the intervention. Considering the data collection challenges posed by the ongoing COVID-19 pandemic, we have developed workarounds to conduct data collection primarily online. We will use systematic online debriefings to facilitate comprehensive participation of the entire research team.

Ethics and Dissemination: Ethical approval has been granted by the Institutional Review Board of the Research Institute for Tropical Medicine (No. 2019-44) and the ethical commission of Heidelberg University, Faculty of Medicine (S-833/2019), both recognized Ethical Review Committees. Study findings will be disseminated in scientific conferences and published in peer-reviewed journals.

Strengths and Limitations of the Study:

- Project SALUBONG directly responds to calls in the literature for more community-based research on how VH can be addressed and how trust in the public health sector can be bolstered.
- Guided by families and communities, we will co-create an empathic intervention that places the health of children, the concerns of parents, and the needs of HCWs at the center to support trust in vaccines.
- Findings will inform future vaccine confidence efforts and contribute to broader policy discussions regarding VH in the Philippines and globally.
- Due to the COVID-19 pandemic, we have shifted some of our data collection online, and ours will be among the first qualitative studies in this setting to outline opportunities and pitfalls of remote qualitative research.

For peer review only - http://bmjopen.bmj.com/site/about/guidelines.xhtml

For peer review only

BACKGROUND

Vaccines are a cost-effective and safe way to prevent millions of deaths annually (1, 2). Although vaccines represent a seminal achievement in terms of mitigating disease, confidence in vaccines has decreased in many countries in recent years (3). This drop in confidence has contributed to stagnation or decreases in immunization rates, which in turn has resulted in outbreaks of previously controlled or eradicated diseases such as measles and polio (4-7).

In 2019, the World Health Organization (WHO) included vaccine hesitancy (VH) - the "delay in acceptance or refusal of vaccines despite availability of vaccination services" (8) - in its list of the top 10 global health threats requiring high-level attention and research (9). Literature that has sought to tease out the causes of VH emphasizes the '5Cs': a lack of confidence (in vaccines and the broader health system), complacency (regarding the severity of vaccine-preventable illness), constraints (psychological, financial or structural barriers), calculation (the degree to which individuals search for information about vaccines), and collective responsibility (a willingness to protect others) (10). More recently, scholars have started to consider the role of trust and unequal power dynamics in undermining vaccine uptake, describing how families have lost trust in the health system and feel that they have no voice in the face of state-mandated decisions or directives about vaccines (11-14).

Effective and efficient solutions to address VH are urgently needed, not only to mitigate the re-emergence of vaccine-preventable diseases (such as polio or measles (6, 15-17)), but also because the development and uptake of an effective vaccine is a cornerstone of controlling the ongoing COVID-19 pandemic (18, 19).

To date there is limited guidance in terms of how to successfully combat VH, most of which stems from high-income settings (20, 21). At the individual level, changing people's attitudes about vaccines has proven difficult, and successful interventions are limited (22). At the governance level, policymakers in high-income countries (HICs) have considered or enacted laws to punish those who reject vaccines (on non-medical grounds) by denying unvaccinated children admission to elementary schools and public playgrounds, and charging parents substantial fines (23). At the health system level, interventions involving medical professionals have considered how to broach the topic of vaccines in a non-judgmental but affirmative manner, how to listen to parents' vaccine decision-making (24) and how to facilitate vaccination directly through reminders, prompts, or by reducing logistical barriers (22).

More recent studies also highlight opportunities in terms of video-based vaccine promotion (24) and educational messages in the form of graphic pictures and anecdotes (focusing on the consequences of not getting a child vaccinated) (25). Several studies have employed Human-Centered Design (HCD), an approach to co-develop interventions with end-users (26). This methodology has led to the creation of mobile apps, education materials, provider guidelines and the re-design of a health facility, all in the interest of bolstering vaccine uptake (24, 27-31). Results of these HCD studies suggest that the approach supports stronger patient and community engagement (24, 27, 30).

For peer review only - http://bmjopen.bmj.com/site/about/guidelines.xhtml

Relatively little attention has been paid to VH and ways to address VH in low- and middle-income countries (LMICs) (14, 21, 24, 25). This is particularly problematic for at least three reasons: 1) a majority of the world's vaccine-preventable deaths occur in LMICs (32); 2) immunization structures in LMICs are insufficiently equipped to address VH *in addition to* rolling out national immunization campaigns (and other child health challenges); and 3) in the event of an outbreak of a vaccine-preventable disease, survival rates and containment possibilities are markedly reduced in LMICs where poor structural conditions and extreme poverty can exacerbate pre-existing vulnerabilities (33-35).

The Philippines, an archipelago with a population of more than 105 million, is among the LMICs that are currently experiencing an unprecedented erosion of public trust in childhood vaccinations (36-38). Vaccine confidence fell from 93% of adults "strongly agreeing" to the importance of vaccination in 2015 to 32% in 2018 (36). This is reflected in measles vaccination rates of children under-five, which fell from 88% in 2014 to 55% in 2018 (39). Results from a recent study conducted in two urban communities in Manila, the capital of the Philippines, showed that 31% of parents feel vaccine hesitant and 24% had refused at least one vaccine for their children (40). These sharp declines are associated with a dengue vaccine controversy in 2017 and the ensuing misinformation that eroded faith in vaccine safety (36-38, 41). This erosion led to the country losing its 19-year polio free status, and sparked measles outbreaks across several islands in 2019, with 47,871 cases including 632 deaths (as compared to 2,789 cases and 25 reported deaths in 2018) (39, 41, 42). The Department of Health (DOH) of the Philippines has therefore made it a priority to win back the trust and confidence of the public in vaccination (43). The DOH - in partnership with the WHO and UNICEF - has strengthened routine immunization via the launch of door-to-door immunization campaigns to increase vaccine uptake and to reach unvaccinated children (16, 44). Although recent data has indicated signs of recovery, with the proportion of people agreeing to the importance of vaccines increasing, gains in terms of perceived vaccine safety and effectiveness are insufficient (3). More tailored and innovative initiatives are necessary to regain community trust in vaccines.

The SALUBONG Intervention

"Salubong", the moniker for this research, is a Filipino term that means to welcome someone into one's home or life. In the Philippines, the largest predominantly Christian country in Asia, the term Salubong describes a Catholic dramatization of the resurrected Jesus encountering his mother Mary, which is a central part of the Easter week across the Philippines. This encounter, which is not liturgically accounted for, culminates in Jesus – if briefly – reuniting with his mother (45). From this, Salubong has developed into a Filipino tradition that celebrates the beauty of re-connecting with important figures from one's past. The Salubong tradition is commonly observed at international airports throughout the Philippines, where many Filipinos are waiting for their loved ones to return home after having sought employment abroad (the diaspora has fostered an undertone of longing and anticipation in many Filipino

For peer review only - http://bmjopen.bmj.com/site/about/guidelines.xhtml

homes) (46, 47). The homecoming of a loved one is viewed as a special and festive event accompanied with a warm embrace and sumptuous meal.

In using this term as our project moniker, we aim to signal to communities that unwelcoming vaccines into homes or lives is a current dilemma, like many of life's dilemmas, but in unwelcoming there is a progression to re-welcoming. Salubong is intertwined with notions of acceptance, compassion and understanding, and sends the message that we will not rely on an intervention that uses the blunt tool of scientific reason or which excludes population perceptions (particularly as this has proven ineffective in relation to VH (25)). Instead, we will employ HCD (48) to co-develop an intervention together with wary families and communities to encourage them to reconsider their views on childhood vaccinations.

Our central thesis is that by drawing from local narratives, designing, refining, and ultimately testing a story-based intervention that bridges families, policymakers, health care workers (HCWs), community leaders and community health workers (following the terminology in the Philippines, where small administrative communities are termed *Barangays*, termed 'barangay health workers' (BHWs) in the context of this study), we will lay the foundation to build a meaningful campaign that revives faith in vaccines. This foundation will contribute to the sustainable prevention of outbreaks of vaccine-preventable diseases.

Study Objective

The purpose of this study is to understand local perspectives of VH in the Philippines, and to develop and pilot a health promotion intervention to address VH. Following the multiple stages of HCD, we will co-design, develop and iterate a community-based intervention (26). The developed intervention will then be piloted to assess effectiveness and acceptability.

Sub-objectives include:

1. To describe family, policymakers and community perceptions of the public health system and vaccines.
2. To gather narratives regarding childhood vaccination and health facility experiences from families, HCWs, BHWs and community leaders (including real-life dialogue between families who delay and refuse vaccinations).
3. To design and pilot a picture or video-based intervention with families and community leaders, and refine it in cooperation with BHWs.

METHODS AND DESIGN

Theoretical underpinning

We draw on the Theory of Planned Behavior (TPB) (49) to acknowledge how control beliefs (whether to vaccinate and the consequences of this decision), attitudes about vaccinations, and normative beliefs (i.e. notions of social responsibility; subjective norms influenced by social or cultural aspects) shape

For peer review only - http://bmjopen.bmj.com/site/about/guidelines.xhtml

the intention to vaccinate and vaccination uptake. Such normative beliefs are of particular relevance in this context, as vaccination uptake has a distinct social responsibility dimension. High vaccination coverage is required to protect those who cannot (yet) get vaccinated, but the possibility of 'freeloading' exists for those who refuse vaccinations due to the risks for the individual, yet can still benefit from others being vaccinated.

A number of studies have employed the TPB to explain VH, supporting its utility and describing potential starting points for interventions (50-52). A recent meta-analysis suggests that the TPB can explain over 50% of the variance in intention to vaccinate, with attitudes and normative beliefs being stronger predictors than perceived behavioral control (53). A 2017 systematic review of trialed vaccine confidence interventions found few interventions with limited efficacy that aimed at changing individuals' attitudes or their awareness of social norms, and instead identified interventions aimed at reducing barriers and therefore increasing behavioral control as a more promising pathway (22).

At the core of the current VH crisis in the Philippines is a recent vaccination controversy, which heavily affected the trust in HCWs, the health system, and other vaccination stakeholders (38). We therefore are also informed by the Socio-Ecological Model (SEM) to understand how vaccination attitudes and behaviors are shaped on individual, interpersonal, organizational, community and public policy levels (54). Kumar and colleagues identified factors across all socio-ecological levels to influence influenza vaccine uptake (55), and a number of authors have argued for including multiple socio-ecological levels when considering how to address VH (56, 57). We will draw on the SEM to gain a better understanding of how stakeholders on different levels perceive barriers or facilitators for vaccinations, and how systematic changes or awareness can increase uptake.

Based on these theoretical models, we developed a framework, which guides this project (**see Figure 1**). With the objective to increase the individual parent's intention to vaccinate their child, we aim at empathetically addressing their attitudes, norms, and perceived behavioral control on an individual level (i.e. the parent receiving the intervention). In developing this intervention, we acknowledge however that factors such as organizational barriers, purveyors of information, or social pressures influence vaccination perception and uptake across several levels of the SEM.

Study setting

The Republic of the Philippines consists of more than 7,000 islands divided into 17 administrative regions (58). This study will be conducted in the Calabarzon region, which has an estimated population of 14 million (of which, 80% accounts for Roman Catholics, and the remainder include Christian denominations and Muslims) including 1.2 million children under age 5; **see Figure 2**) (59). Calabarzon experienced a 300% increase in measles cases in 2019 as compared to 2018 (39, 60, 61). The region is middle-income and predominantly consists of agriculture, fishing, manufacturing and high technology industries (62, 63). Calabarzon is composed of five provinces, 20 cities, 123 municipalities and 4,018 *Barangays* ("small communities"). The project will be conducted in Dasmariñas City and rural

For peer review only - http://bmjopen.bmj.com/site/about/guidelines.xhtml

municipalities of Cavite province, which were purposively selected to reflect both rural and urbanized conditions, and to capture varied socio-demographic factors and health facility related experiences in terms of child health and vaccinations.

Study Population

Our study population will include community leaders (such as the *Barangay* Captains and Councilors on Health), families of young children, policymakers, HCWs (municipal/city health officers, nurses, and midwives), and BHWs (community-based health volunteers who help and assist nurses and midwives in the delivery of essential health care programs (64)). Ethnicity, race, political orientation, religion and class are not criteria for inclusion or exclusion in this study. Respondents will be eligible to participate in the study if they live within Cavite Province and Dasmariñas City. Respondents must be at least 18-years-old or an emancipated minor (who are 15-17 years old but with children under-5) to participate. Incapacitated persons are excluded.

Study Design

Informed by HCD (26), this study employs a mixed methods, exploratory sequential design, drawing on qualitative and then quantitative methods (65). We will first use qualitative methods (IDIs and FGDs) and then quantitative methods (pre-post surveys and Kano questionnaires). **Figure 3** shows the summary of the five study phases (a preparatory phase followed by the four phases of HCD), along with specific objectives and expected corresponding outputs. Within any given phase, iteration and repetition is likely and usually necessary. The ultimate goal of the process is to ensure that the fundamental design of a product or program reflects what users want and works in the setting where they will be using it.

1. **Preparatory phase.**

We will conduct IDIs with policymakers (n=15-20) to understand the current challenges for ongoing public health efforts on childhood vaccinations, perceptions of vaccines and the health system. Further, we will also explore how the current COVID-19 pandemic poses challenges and opportunities to health education and vaccination efforts.

2. **Phase 1. Shared Appraising (EMPATHIZE PHASE):**

This phase of HCD aims at gathering information about how users frame a childhood vaccination problem, how they situate themselves in relation to the problem (probing on socio-cultural context) and learning which factors would motivate them to address the problem.

IDIs with parents of children under five: We will conduct IDIs (n=60) with parents of children under five. In each selected *barangay* in Cavite province and Dasmariñas city, we will purposively select 30 parents recommended by a nurse or midwife who will have reviewed vaccination records in order to identify families that have delayed or refused childhood vaccines. Based on IDIs, we will build holistic

For peer review only - http://bmjopen.bmj.com/site/about/guidelines.xhtml

narratives about family perceptions of the public health system and encounters with childhood vaccinations. Participants with particular vaccination or VH experiences will be asked if they are willing to be video-recorded as they tell their personal story related to vaccines. These video-recorded interviews will aid pre-development of the intervention.

IDIs with HCWs and community leaders: IDIs (n=10) will also be conducted among HCWs (municipal/city health officers, nurses, midwives) and community leaders (specifically the *Barangay* Captain and Councilors for Health) of the selected *barangays* to describe their experiences related to childhood vaccination in their respective community or health facility. Following each interview, the preliminary storyboards developed as a result of the initial IDIs (i.e. 10-15 IDIs) will be presented to participants. Participants (parents, HCWs) will be asked to conduct a think-aloud exercise while flipping through these storyboards. After each 10-15 IDIs, or at the end of each week of data collection, the storyboards will be edited and refined based on participants' comments. These revised storyboards will be included in the following round of IDIs for further refinement.

3. **Phase 2. Life stories and Uncovering (DEFINE & IDEATE PHASE):**

In this phase, end-users will suggest ideas to address the problem in collaboration with a research team.

Records Review: From the selected *barangays*, the research team will seek the help of BHWs to identify potential participants based on childhood vaccination records. We will purposively select 50 potential participants in each selected *barangay* of Cavite province and Dasmariñas City and review their vaccination record through Target Client Lists (TCL) and Individual Treatment Records (ITRs). Each case will be allocated to one of three categories:

1. *Fully Immunized Child (FIC):* Children who complete one dose of BCG, three doses of Oral Polio Vaccine (OPV), three doses of Diptheria-Pertussis-Tetanus (DPT), three doses of Hepatitis B and 1 dose of Measles Vaccine BEFORE a child's first birthday. These are the children who received their vaccinations within the National Immunization Program (NIP) schedule.
2. *Completely Immunized Child (CIC):* Children who receive one dose of BCG, three doses of OPV, three doses of DPT, three doses of Hepatitis B and one dose of Measles Vaccine AFTER a child's first birthday. These are children who have delays in receiving vaccination based on the recommended NIP schedule.
3. *Refusal to vaccinate:* Children with the remark "refused" in the TCL will be cross-validated with the respective ITR to further review their reasons of refusal.

This process will allow us to initially stratify the participants for FGDs based on their children's vaccination status, preventing contamination and conflict during discussions. Additionally, we will use the records review to confirm delays in children's vaccination schedules, even if these children were later fully immunized.

For peer review only - http://bmjopen.bmj.com/site/about/guidelines.xhtml

Focus Group Discussions: We will conduct FGDs (n=10-15) with parents, stratified (based on the records review and validation of child vaccination cards) into three groups by their views: 1) in favor, 2) delay and 3) refusal of vaccination, to understand how these views evolved or persisted. We will explore the socially held attitudes toward the public health system and vaccines. We will also conduct unstratified FGDs among BHWs of the sampled *barangays* to understand their community and health facility experiences on childhood vaccinations. Results of these FGDs will inform the development of storyboard flip-boards that will be refined in the next phases of the project. To ensure that the design itself of the intervention resonates with the intricate details of Filipino demographic and cultural dynamisms, we collaborate with local animators with years of groundwork experience in developing interventions in the Philippines who will accompany FGDs and graphically record ideas and concepts in real-time, which can be discussed among participants.

4. **Phase 3. Bridging and Optimizing (PROTOTYPE PHASE):**

In this phase, prototypes and products are developed and tested in real-world settings with actual users via actual delivery systems.

IDIs with HCWs and BHWs: The draft intervention, together with data from our FGDs and IDIs will be presented to HCWs (municipal/city health officers, nurses, midwives) and BHWs. We will conduct 10 IDIs with HCWs and BHWs to get their perspectives and recommendations regarding additional information that needs to be included or refined, namely related to how, when and by whom the intervention should be delivered. These IDIs will guide the research team in terms of preferred medium (e.g. paper versus video-based presentation) and favorable delivery approach (e.g. spoken text to accompany the intervention, one-on-one vs. group delivery, a stand-alone activity vs. nested in existing outreach, etc.). The result of this phase will allow us to further refine and finalize the intervention, and to determine the "point of contact" for the delivery of the intervention in Phase 4.

5. **Phase 4. Navigating and Gaining (TESTING PHASE)***:*

In this phase, the intervention is introduced to and tested with a larger sample, and large-scale feedback is sought.

Pilot Testing: We will test the chosen model (intervention + point of contact) in 20-30 *barangays* (divided into urban and rural areas) selected in the previous phases. **Figure 4** shows the detailed design of the pilot phase. The research team will identify and train the BHWs as "champions" of childhood vaccination, an approach that has been critical in the success of several maternal and child health programs in the sub-Saharan Africa (66). These champions will then deliver the intervention in specific "points of contact" (e.g. one-on-one vs. group delivery of the videos; a stand-alone activity vs. nested in existing outreach) as determined in Phase 3. Before and after the BHW delivers the intervention, the study team will administer short surveys. The pre- and post-intervention surveys (based on best practices for measuring VH (8, 67)) will quantify participants' knowledge, attitudes, and practices

For peer review only - http://bmjopen.bmj.com/site/about/guidelines.xhtml

regarding vaccination and VH, and the post-intervention survey will additionally assess any changes in knowledge or attitude. We will revise the survey tool based on outcome from phases one and three.

Intervention Feedback: After pilot testing, 200 participants (100 participants randomly sampled from each the urban and rural arms) will be asked a short series of closed-ended questions about features of the intervention and feedback on how the intervention made the user feel based on Kano analysis methodology (attractive vs. essential) (68). After the Kano survey, 25 participants in each area (n=50) will be purposively selected based on critical case sampling and invited to an FGD (n=3-5) to identify comprehension problems and to seek further feedback in smaller groups. Since this study only aims to pilot test the developed intervention, the feedback on the intervention will be useful for further refinement before future trials.

Patient and Public Involvement

Neither clients nor the public were directly involved in the design, recruitment nor conduct of the study; their only involvement is as research participants. However, following the tenets of HCD, the intervention is co-developed with research participants over the entire course of the study. Participants are not involved in initial recruitment, but those participants completing an IDI may be asked to provide contact details of others who might be interested to participate in the study. Participants are not involved in data analysis or the dissemination of findings. The final results will be shared with policy makers, health programmers, and other stakeholders via roundtable discussion, and in the form of articles published in peer-reviewed journals or policy briefs. Research participants will be given access to the final intervention and be informed through their BHWs and other channels embedded in the community, and have the option to contact study staff for a detailed description of study findings at any time.

Sample Sizes and Sampling Technique

We will partner with HCWs and BHWs who keep lists of mothers of young children to identify eligible households, particularly families with various experiences or perspectives on vaccines in order to help us capture a range of insights in terms of vaccine attitudes and behaviors.

Qualitative component: Purposive sampling will be utilized for the qualitative components of the Pre-phase and Phases 1-3 to gain maximum range of perspectives and depth of information; sample size estimates are guided by saturation estimates and outlined in **Figure 1** (69, 70).

Quantitative component: During Phase 4 we will rely on household survey data. The estimated sample size per group (urban and rural arms) is 300, which will result in a total of 600 survey respondents, which offers a binary outcome margin of error of +/- 7% within each group at a 95% confidence level, and a design effect of 1.5. To select the study sites, a multi-stage stratified random sampling frame will be used. The sampling scheme is illustrated in **Figure 5**.

From each selected area in Dasmariñas City and in a rural municipality in Cavite, 10-15 *barangays* will be selected purposively: All *barangays* will be ranked from highest to lowest number

For peer review only - http://bmjopen.bmj.com/site/about/guidelines.xhtml

of annual births in the most recent report available (preferably 2018-2019) in the Rural Health Unit/City Health Office, and the cumulative sum of the number of births will be computed. We will conduct the survey in those *barangays*, which reported at least 300 births, or in a maximum of 15 *barangays* with the highest number of births, whichever threshold is reached first.

In each of the *barangays* to be surveyed, a list of the mothers with children under one year old will be obtained from the midwife or the BHWs. All mothers listed will be invited to participate in the study. In the event that the number of interviewed mothers is less than 300, the survey will be continued in the next *barangay* on the list.

Data Collection and Data Collector Training

Before the onset of data collection, we will contact Department of Health (DOH) officials (national, regional and provincial offices) to explain study objectives and procedures, and to discuss and resolve any concerns regarding data collection. We will also seek their permission and support to conduct the project in the selected primary health care facilities. Official communications explaining the nature, study objectives and procedures will be sent to local government executives and community leaders. Spot maps of the community, if available, will be requested from the Rural Health Units or community health centers. The spot maps will be used to locate prospective families in communities. Courtesy calls to local officials and leaders will be made to seek their support to conduct the project in their respective localities.

The research team will conduct the qualitative interviews, FGDs and quantitative surveys with the assistance of field interviewers (FIs) in either English or Filipino, depending on the preference of the participant. FIs will be fluent in English and Filipino with bachelor's level education in nursing, midwifery or social sciences. FIs will be trained for five days to collect the data with instruments and online platforms for data collection for this study. Training topics will include modules on VH, interviewing and/or surveying techniques, research ethics, software resources, Kano analysis and qualitative and quantitative methods.

Data Collection in times of Covid-19

All IDIs and FGDs were initially planned to be conducted in-person. However, the ongoing pandemic and measures taken to curb infection rates pose unique challenges to conducting research. Considering the time-sensitive nature of this project, we have developed new operating procedures for conducting online data collection, to ensure minimal risk for participants and the research team concerning COVID-19, and in compliance with the recommendations by the European Medicines Agency (71) and the Philippines Inter-Agency Task Force (IATF) (72). This includes informed consent processes and the engagement of research team members who are unable to travel to the study site. Until the situation changes, only strictly necessary visits will be performed at sites.

For peer review only - http://bmjopen.bmj.com/site/about/guidelines.xhtml

Remote recruitment and consenting: We will contact prospective participants via email or a phone call to briefly introduce the study aims and invite them to participate. After this initial phone call or email, we will set an appointment for the comprehensive discussion of the study and to answer questions. An information sheet (including study aims, procedures, and expected risks and benefits) and consent forms will be sent to potential respondents in advance via email or courier. If participants agree to enroll in the study, we will ask them to sign the consent form during a recorded Skype or Zoom video call, and to take a picture while holding the signed consent form. The signed consent forms will be returned to the study team as scanned or photographed copy, or per courier (as preferred by the participant; courier costs will be covered by the study team). Participants will receive duplicate copies with the signature of the interviewer per courier.

Focus Group Discussions: At the time of writing (October 2020), we continue to hope that by the beginning of 2021, when we plan to conduct FGDs, small group discussions in person with appropriate distance will be possible. However, we will conduct remote FGDs if necessary, to ensure the safety of participants and research team. In this case, we will draw on the assistance of local stakeholders (HCWs, BHWs etc.) to orient participants on group discussions via a web-based platform.

Systematic Debriefings: As a number of research team members are not be able to travel to the study site due to Covid-19 travel restrictions, we will employ remote systematic debriefings (73) to discuss and triangulate findings, to amend interview guides and to refine lines of inquiry.

DATA PROTECTION AND ANALYSIS

Data quality checks and cleaning process

Most of the data in the project will be primary and collected by the research team. Interviews will be audio-recorded using digital or online audio-recorders. All recorded information will be transcribed verbatim and translated into English. For all qualitative data, the research team collecting and analyzing the qualitative data will be directly responsible for quality checks of audio recordings, interview notes and qualitative transcripts. Random checks of transcripts will be conducted to support quality.

All quantitative data will be entered into a customized Microsoft Access data entry system with a built-in validation program. The data management unit (DMU) of the Research Institute for Tropical Medicine (RITM)'s Department of Epidemiology and Biostatistics (DEBS) will be responsible for quality checks. DMU staff will reach out to the SALUBONG team in the event of questions or data inconsistencies.

Data storage and protection

Both primary and secondary data (quantitative and qualitative) collected by the research team will be stored according to German and Philippine regulations. At the point of data collection, unique codes will be assigned to all participants. A master sheet linking the code with identifying information (including a name and, when feasible, contact information) will be kept in a secure (locked/password

For peer review only - http://bmjopen.bmj.com/site/about/guidelines.xhtml

protected) location that is accessible to the investigators. Only the research coordinator will have access to and manage this master sheet - across quantitative and qualitative data. The master sheet will facilitate the process of revoking information should a respondent decide (at a later point) that they wish to have their data removed from the study. Further, audiotaped interviews will also be stored in locked/password-protected computers controlled by the research coordinator. All data will be accessible only to those within the research team, but can be made available to others upon reasonable request after approval from ethical review boards.

Data analysis

While the work is divided into phases, the ultimate aim of this study is to analyze and apply findings continuously across phases to address the overall study objective. The PIs and Co-Is will be directly responsible for the triangulation of findings across data sources and the development of an integrated interpretative analysis.

Qualitative analysis

The qualitative component of this study will be analyzed based on constructivist grounded theory as outlined by Charmaz (74). The application of this approach will help us generate theories on why parents would or would not agree to childhood vaccinations, and it will allow us to investigate the constructions and underlying processes of parents' perceptions, so that informed and relevant approaches can be applied to help resolve highly salient concerns. NVivo Pro 12 (QSR International Pty Ltd. Version 12, 2018) will support qualitative analysis.

Quantitative Analysis

The quantitative component of this study will be analyzed using descriptive and inferential approaches, facilitated by Stata statistical software (*Stata Statistical Software: Release 13.* College Station, TX: StataCorp LP). The effect of the intervention on the knowledge, attitude and practices scores will be analyzed using *t*-test inferential statistics. Knowledge and practices scores will be calculated using a point marking system, (1 point for each correct and 0 for each false answer or no answer). Further, we will calculate whether knowledge has increased in total (i.e. the total points in the suggested approach are higher pre- versus post-intervention) and in which particular domain knowledge has increased. Items on attitudes (Likert scale) will be analyzed using descriptive statistics. Additionally, the Likert scale will be analyzed inferentially by coding answers from 1 (strongly disagree) to 5 (strongly agree) and drawing on parametrical (paired *t*-test) or non-parametrical (*Wilcoxon signed rank test*) approaches, depending on sample characteristics.

Kano-style questions place user ratings of product or service features on a two-dimensional scale (38). One dimension is satisfaction, which can range from frustration to delight. The other is functionality, which ranges from not functional to highly effective. Hence, Kano analysis of post-

For peer review only - http://bmjopen.bmj.com/site/about/guidelines.xhtml

intervention survey responses to Kano-style questions will be used to prioritize resource allocation in improving the intervention functionality, with the intention of causally affecting customers' satisfaction (38). The results will then be used for the revision or re-development of intervention features for future, large-scale testing.

DISCUSSION

Although vaccination uptake and VH are central parts of the current public health and global health discourses, few interventions have proven efficacious in sustainably increasing vaccine confidence. In this study protocol, we explain how we will work with clients, providers and policymakers to develop a narrative-based intervention to increase vaccine confidence in the Philippines. Guided by the tenets of HCD, we will co-develop and iterate this intervention to address the needs and expectations of those it is developed to support.

HCD is a promising new approach in the development of global health interventions (48). In our application of this methodology we will move beyond the way it currently is often applied and extend the understanding of 'clients' (following terminology from HCD field understood here as the end-users of the intervention) to those involved across several levels including: parents who are deciding whether to vaccinate their children (clients in the sense of 'target population'), HCWS and BHWs who interact with parents (clients in the sense of those who will – or will not – incorporate the intervention into their service delivery) and policymakers and key vaccination stakeholders whose decisions and policies shape the vaccination landscape (clients in the sense of those who decide the implementation of large scale interventions).

We also expand the existing body of literature on HCD-based vaccine confidence interventions by drawing on a theoretical framework to inform our sampling and design procedures and using well-established behavior change theories to guide data collection activities. This for example includes identifying topics to be probed for in IDIs (e.g. perceived behavioral control about getting a child vaccinated), but also those topics with a clearly social dimension to be focused on in FGDs (e.g. social responsibility of vaccinations). We therefore do not employ a purely inductive approach for conducting qualitative research, as is common in global health qualitative research and HCD approaches, but allow for the existing literature and established theories to guide our approach.

Previous studies demonstrate that decision-making surrounding vaccine is complex, and that those who provide vaccine-related information are in a unique position to sway or break vaccine acceptance (75, 76). A high general trust in the health authorities and providers that decide what vaccines to introduce is a deciding factor in vaccine uptake (77). However, trust and source credibility plays a pivotal role not only in direct communication between healthcare providers and their clients, but also across all levels: Health-related controversies have been shown to disrupt the trust healthcare providers have in political agents, or implementing government institutions have in those who make the respective

For peer review only - http://bmjopen.bmj.com/site/about/guidelines.xhtml

policies (38). Our inclusion of stakeholders from all levels in the design process will support our ability to identify pathways to rebuild both confidence in vaccines and trust in the general system.

The Dengvaxia-controversy in the Philippines highlights the pace and reach of misinformation regarding the effectiveness and safety of a vaccine, causing a drop in general vaccine confidence and rolling back decades of immunization work (36, 38). Dayrit and colleagues (38) stated that the communication gaps among the health authorities themselves, as well as a lack of transparency, proliferation of non-scientific speakers in the mainstream media, and distorted messages in social media played a key role in the panic felt among Filipino families. The Dengvaxia controversy was exacerbated by an exceptionally rapid spread of (mis)information via social media channels (36, 38). In the broader context of vaccination, factually false or misleading information shared in online forums or via social media, often in the form of short quotes or emotionalized images, is considered a central contributor to anti-vaccination narratives (78). In many cases, countering these narratives is challenging as it requires longer and complex explanations of vaccination risks and benefits, which are not as easily spread through social media (79, 80). We are taking this into consideration while developing our narrative-based intervention in part or entirely in a form that can be easily relayed (e.g. as a short video or even a GIF). With vaccine confidence or uptake interventions delivered via social media showing promising results (81, 82), we explicitly include this notion in our HCD-based discussion of promising delivery strategies.

The Dengvaxia controversy has been repeatedly linked to the steep decline in vaccination rates and vaccine confidence in the Philippines (3, 36). However, to the best of our knowledge, there so far has not been any qualitative exploration of how this case has shaped narratives about vaccines, health programming and the health system in general. Furthermore, there has been remarkably little research highlighting Philippine perspectives on how to move forward after the controversy. The SALUBONG project will thus provide robust, culturally-attuned data that can inform future programs and policies to rebuild trust and confidence in relation to vaccines (22, 83, 84).

Finally, similar to many research projects, the ongoing COVID-19 pandemic has forced us to reconsider the way we collect data to ensure the safety of our participants and study team. We have developed new approaches to remotely undertake this study. In close cooperation with the ethical review boards evaluating this project, we have developed protocols to ensure that recruitment, consent and rapport building are supported despite the remote nature of data collection. We hope that the procedures outlined in this protocol will spark a discourse on how to conduct ethical and trustworthy research during a pandemic, and that our experiences will allow us to further develop and validate methodological approaches.

DECLARATIONS

For peer review only - http://bmjopen.bmj.com/site/about/guidelines.xhtml

Acknowledgements: We would like to thank Prof. Dr. Manuela de Allegri and Dr. Veronica Tallo for their expert advice on methodology and local study implementation. Special appreciation is also given to Meghan Obermeyer for her inspiring global health stories, which sparked the idea of this project.

Contributors: MDR participate in all aspects of the study design, set up of local study implementation systems and led the writing of the protocol. JW oversaw the Heidelberg ethics approval process, contributed to and edited the protocol paper. KB participated in the design and writing of the qualitative components and edited the protocol paper. MA, JLG, VE, JL, TAB, MPD, JRG and NDC participated setting up local implementation systems including development of standard operating procedures for online data collection and edited the protocol paper. MT advised on the quantitative study design, sample size calculation, local study implementation and edited the protocol paper. MA advised on the HCD process and edited the protocol paper. RC advised on the quantitative study design, sample size calculation and edited protocol paper. SM supervised all aspects of the study design, the writing and editing of the protocol paper. All authors have read and approved the final version of the manuscript.

Funding: This study has been externally reviewed and funded following a competitive grant application and review process by the Global Grand Challenges, Bill and Melinda Gates Foundation [OPP1217275]. Under the grant conditions of the Foundation, a Creative Commons Attribution 4.0 Generic License has already been assigned to the Author Accepted Manuscript version that might arise from this submission. The funders do not have competing interests and did not play a role in the preparation of this protocol paper.

Competing interests: The authors declare no competing interests. The content is the responsibility of the authors and does not necessarily represent the views of any funder.

Availability of data and materials: Not applicable.

Patient consent for publication: Not applicable. (This manuscript does not contain any identifiable data.)

Provenance and peer review: Not commissioned; externally peer reviewed

Open access: This is an open access article distributed in accordance with the Creative Commons Attribution Non Commercial (CC BY-NC 4.0) license, which permits others to distribute, remix, adapt, build upon this work non-commercially, and license their derivative works on different terms, provided the original work is properly cited, appropriate credit is given, any changes made indicated, and the use is non-commercial. See: http://creativecommons.org/licenses/by-nc/4.0/.

For peer review only - http://bmjopen.bmj.com/site/about/guidelines.xhtml

REFERENCES

1. Omer S, Salmon D, Orenstein W, deHart P, Halsey N. Vaccine Refusal, Mandatory Immunization, and the Risks of Vaccine-Preventable Diseases. N Engl J Med. 2009;360:1981-8.
2. WHO. Immunization coverage. 2020.
3. de Figueiredo A, Simas C, Karafillakis E, Paterson P, Larson HJ. Mapping global trends in vaccine confidence and investigating barriers to vaccine uptake: a large-scale retrospective temporal modelling study. Lancet. 2020.
4. Patel MK, Orenstein WA. Classification of global measles cases in 2013-17 as due to policy or vaccination failure: a retrospective review of global surveillance data. Lancet Glob Health. 2019;7(3):e313-e20.
5. Principi N, Esposito S. Early vaccination: a provisional measure to prevent measles in infants. Lancet Infect Dis. 2019;19(11):1157-8.
6. WHO. European Region loses ground in effort to eliminate measles. . Copenhagen2019.
7. Department of Health. Polio Case Confirmed in the Philippines: DoH To Mount Mass Immunization Campaign Philippines: Department of Health; 2019 [Available from: https://www.doh.gov.ph/node/18012.
8. Group WSW. Report of the SAGE Working Group on Vaccine Hesitancy.; 2014.
9. WHO. Ten threats to global health 2019 2019 [Available from: https://www.who.int/emergencies/ten-threats-to-global-health-in-2019.
10. Betsch C, Schmid P, Heinemeier D, Korn L, Holtmann C, Bohm R. Beyond confidence: Development of a measure assessing the 5C psychological antecedents of vaccination. PLoS One. 2018;13(12):e0208601.
11. Lane S, MacDonald NE, Marti M, Dumolard L. Vaccine hesitancy around the globe: Analysis of three years of WHO/UNICEF Joint Reporting Form data-2015-2017. Vaccine. 2018;36(26):3861-7.
12. Marti M, de Cola M, MacDonald NE, Dumolard L, Duclos P. Assessments of global drivers of vaccine hesitancy in 2014-Looking beyond safety concerns. PLoS One. 2017;12(3):e0172310.
13. Larson H. Stuck: How Vaccine Rumors Start — and Why They Don't Go Away: Oxford University Press; 2020.
14. Dube E, Gagnon D, MacDonald NE, Hesitancy SWGoV. Strategies intended to address vaccine hesitancy: Review of published reviews. Vaccine. 2015;33(34):4191-203.
15. Benecke O, DeYoung SE. Anti-Vaccine Decision-Making and Measles Resurgence in the United States. Glob Pediatr Health. 2019;6:2333794X19862949.
16. WHO. Immunization gaps contribute to rising measles cases in the Philippines Philippines2019 [Available from: https://www.who.int/philippines/news/feature-stories/detail/immunization-gaps-contribute-to-rising-measles-cases-in-the-philippines.
17. The Lancet Infectious D. Infectious disease crisis in the Philippines. Lancet Infect Dis. 2019;19(12):1265.
18. Mello MM, Silverman RD, Omer SB. Ensuring Uptake of Vaccines against SARS-CoV-2. N Engl J Med. 2020;383(14):1296-9.
19. Schaffer DeRoo S, Pudalov NJ, Fu LY. Planning for a COVID-19 Vaccination Program. JAMA. 2020;323(24):2458-9.
20. Sadaf A, Richards JL, Glanz J, Salmon DA, Omer SB. A systematic review of interventions for reducing parental vaccine refusal and vaccine hesitancy. Vaccine. 2013;31(40):4293-304.
21. Jarrett C, Wilson R, O'Leary M, Eckersberger E, Larson HJ, Hesitancy SWGoV. Strategies for addressing vaccine hesitancy - A systematic review. Vaccine. 2015;33(34):4180-90.
22. Brewer N, Chapman G, Rothman A, Leask J, Kempe A. Increasing Vaccination: Putting Psychological Science Into Action. Psychological Science in the Public Interest. 2017;18(3):149-207.
23. Tanne JH. US county bars unvaccinated children from public spaces amid measles emergency. BMJ. 2019;364:l1481.
24. Chamberlain AT, Limaye RJ, O'Leary ST, Frew PM, Brewer SE, Spina CI, et al. Development and acceptability of a video-based vaccine promotion tutorial for obstetric care providers. Vaccine. 2019;37(19):2532-6.
25. Horne Z, Powell D, Hummel JE, Holyoak KJ. Countering antivaccination attitudes. Proc Natl Acad Sci U S A. 2015;112(33):10321-4.
26. Bazzano AN, Martin J, Hicks E, Faughnan M, Murphy L. Human-centred design in global health: A scoping review of applications and contexts. PloS one. 2017;12(11):e0186744.

For peer review only

For peer review only - http://bmjopen.bmj.com/site/about/guidelines.xhtml

27. Seeber L, Conrad T, Hoppe C, Obermeier P, Chen X, Karsch K, et al. Educating parents about the vaccination status of their children: A user-centered mobile application. Prev Med Rep. 2017;5:241-50.
28. Henninger ML, McMullen CK, Firemark AJ, Naleway AL, Henrikson NB, Turcotte JA. User-Centered Design for Developing Interventions to Improve Clinician Recommendation of Human Papillomavirus Vaccination. Perm J. 2017;21:16-191.
29. Minoletti S, Rapisarda R, Giraldo L, Grande M, Sommer J, Plazzotta F, et al. User-Centered Design of a Pediatric Vaccination Module for Patients. Stud Health Technol Inform. 2019;264:1096-100.
30. Seeber L, Michl B, Rundblad G, Trusko B, Schnjakin M, Meinel C, et al. A design thinking approach to effective vaccine safety communication. Curr Drug Saf. 2015;10(1):31-40.
31. Inc. JS. VxDel Data Research: Kenya Initial Insights. 2020.
32. Enhancing sustainable access to vaccines for populations in Middle Income Countries (MICs) [Internet]. 2015. Available from: https://www.who.int/immunization/programmes_systems/sustainability/mic_strategy/en/.
33. Muñoz DC, Llamas LM, X. B-C. Exposing concerns about vaccination in low- and middle-income countries: a systematic review. International Journal of Public Health. 2015;60:767-80.
34. Madhav N, Oppenheim B, Gallivan M, Mulembakani P, Rubin E, Wolfe N. Pandemics: Risks, Impacts, and Mitigation. In: rd, Jamison DT, Gelband H, Horton S, Jha P, Laxminarayan R, et al., editors. Disease Control Priorities: Improving Health and Reducing Poverty. Washington (DC)2017.
35. Turner HC, Thwaites GE, Clapham HE. Vaccine-preventable diseases in lower-middle-income countries. Lancet Infect Dis. 2018;18(9):937-9.
36. Larson HJ, Hartigan-Go K, de Figueiredo A. Vaccine confidence plummets in the Philippines following dengue vaccine scare: why it matters to pandemic preparedness. Hum Vaccin Immunother. 2019;15(3):625-7.
37. Fatima K, Syed NI. Dengvaxia controversy: impact on vaccine hesitancy. J Glob Health. 2018;8(2):010312.
38. Dayrit MM, Mendoza RU, Valenzuela SA. The importance of effective risk communication and transparency: lessons from the dengue vaccine controversy in the Philippines. J Public Health Policy. 2020;41(3):252-67.
39. Philippines U-W. Situation Report 3: Measles Outbreak. . Philippines: World Health Organization Philippines; 2019.
40. Migriño J, Gayados B, Birol K, De Jesus L, Lopez C, Mercado W, et al. Factors affecting vaccine hesitancy among families with children 2 years old and younger in two urban communities in Manila, Philippines. Western Pacific Surveillance and Response Journal. 2020;11(2).
41. Dyer O. Philippines measles outbreak is deadliest yet as vaccine scepticism spurs disease comeback. BMJ. 2019;364:l739.
42. IFRC. Operations Update Philippines: Re-emergence of vaccine preventable diseases – Measles outbreak. International Federation of Red Cross and Red Crescent Societies; 2020.
43. DOH identifies Vaccine Hesitancy as one of the reasons for measles outbreak [press release]. Manila, Philippines: Department of Health.
44. UNICEF. Turning doubters into defenders to fight a measles outbreak in the Philippines Philippines2019 [Available from: https://www.unicef.org/philippines/stories/turning-doubters-defenders-fight-measles-outbreak-philippines.
45. Fernandez D. Salubong in Angono, Rizal. Philippine Studies. 1980;28(2):212-20.
46. Alburo J. Boxed In or Out? Balikbayan Boxes as Metaphors for Filipino American (Dis)Location. Ethnologies. 2020;27(2):137-57.
47. Blanc CS. Balikbayan: A Filipino Extension of the National Imaginary and of State Boundaries. Philippine Sociological Review. 1996;44(1):178-93.
48. IDEO.org. The field guide to human-centered design: design kit2015.
49. Fishbein M, Ajzen I. Predicting and Changing Behavior: The Reasoned Action Approach. New York: Psychology Press; 2010.
50. Fahy A, Desmond DM. Irish mothers' intentions to have daughters receive the HPV vaccine. Ir J Med Sci. 2010;179(3):427-30.
51. Malo TL, Hall ME, Brewer NT, Lathren CR, Gilkey MB. Why is announcement training more effective than conversation training for introducing HPV vaccination? A theory-based investigation. Implement Sci. 2018;13(1):57.
52. Britt RK, Hatten KN, Chappuis SO. Perceived behavioral control, intention to get vaccinated, and usage of online information about the human papillomavirus vaccine. Health Psychol Behav Med. 2014;2(1):52-65.

For peer review only - http://bmjopen.bmj.com/site/about/guidelines.xhtml

53. Xiao X, Wong RM. Vaccine hesitancy and perceived behavioral control: A meta-analysis. Vaccine. 2020;38(33):5131-8.
54. NIH NIoH. Theory at a Glance: A Guide for Health Promotion Practice (Second Edition). 2005.
55. Kumar S, Quinn SC, Kim KH, Musa D, Hilyard KM, Freimuth VS. The social ecological model as a framework for determinants of 2009 H1N1 influenza vaccine uptake in the United States. Health Educ Behav. 2012;39(2):229-43.
56. Kolff CA, Scott VP, Stockwell MS. The use of technology to promote vaccination: A social ecological model based framework. Hum Vaccin Immunother. 2018;14(7):1636-46.
57. Downs LS, Jr., Scarinci I, Einstein MH, Collins Y, Flowers L. Overcoming the barriers to HPV vaccination in high-risk populations in the US. Gynecol Oncol. 2010;117(3):486-90.
58. Philippines Statistics Office P, ICF. Philippines National Demographic and Health Survey 2017. Quezon City, Philippines, and Rockville, Maryland, USA: PSA and ICF.; 2018.
59. Regional Socio-Deomgraphic Profile [Internet]. [cited October 1, 2020]. Available from: https://rpo4a.popcom.gov.ph/2015-05-26-08-42-52/2015-05-26-09-21-46?showall=1.
60. Services O. Philippines: Measles Outbreak - Feb 2019 2019 [Available from: https://reliefweb.int/disaster/ep-2019-000112-phl.
61. News O. Measles: Calabarzon reports 3,835 percent increase in 2019. Outbreak News Today. 2019.
62. Group OB. Calabarzon a key industrial region, adding significantly to the Philippines' GDP [Available from: https://oxfordbusinessgroup.com/analysis/surpassing-expectations-calabarzon-key-industrial-growth-region-contributing-significantly-national.
63. EMB EMB. CALABARZON [Available from: http://calabarzon.emb.gov.ph/about-us/.
64. Yamashita T, Suplido SA, Llave C, Tuliao MT, Tanaka Y, Matsuo H. Understanding Postpartum Healthcare Services and Exploring the Challenges and Motivations of Maternal Health Service Providers in the Philippines: a Qualitative Study. Trop Med Health. 2015;43(2):123-30.
65. Creswell J. Research Design: Qualitative, Quantitative, and Mixed Methods Approaches. 4th ed. United States of America: Sage Publications; 2014.
66. Dao B, Otolorin E, Gomez PP, Carr C, Sanghvi H. Preparing the next generation of maternal and newborn health leaders: the maternal and newborn health champions initiatives. Int J Gynaecol Obstet. 2015;130 Suppl 2:S40-5.
67. Larson HJ, Jarrett C, Schulz WS, Chaudhuri M, Zhou Y, Dube E, et al. Measuring vaccine hesitancy: The development of a survey tool. Vaccine. 2015;33(34):4165-75.
68. Lin FH, Tsai SB, Lee YC, Hsiao CF, Zhou J, Wang J, et al. Empirical research on Kano's model and customer satisfaction. PLoS One. 2017;12(9):e0183888.
69. Dicicco-Bloom B, Crabtree BF. The qualitative research interview. Med Educ. 2006;40(4):314-21.
70. Vasileiou K, Barnett J, Thorpe S, Young T. Characterising and justifying sample size sufficiency in interview-based studies: systematic analysis of qualitative health research over a 15-year period. BMC Med Res Methodol. 2018;18(1):148.
71. Agency EM. Guidance on the management of clinical trials during the COVID-19 (Coronavirus) Pandemic 2020.
72. Force I-AT. Inter-Agency Task Force for the management of emerging infectious diseases: Resolution No. 69-A. Manila, Philippines: Official Gazette; 2020.
73. McMahon SA, Winch PJ. Systematic debriefing after qualitative encounters: an essential analysis step in applied qualitative research. BMJ Glob Health. 2018;3(5):e000837.
74. Charmaz K. Constructing Grounded Theory: A practical guide through qualitative analysis. David S, editor. London, Great Britain: SAGE Publications Ltd; 2006.
75. Greenberg J, Dube E, Driedger M. Vaccine Hesitancy: In Search of the Risk Communication Comfort Zone. PLoS Curr. 2017;9.
76. Jennings FJ, Russell FM. Civility, credibility, and health information: The impact of uncivil comments and source credibility on attitudes about vaccines. Public Underst Sci. 2019;28(4):417-32.
77. Freed GL, Clark SJ, Butchart AT, Singer DC, Davis MM. Sources and perceived credibility of vaccine-safety information for parents. Pediatrics. 2011;127 Suppl 1:S107-12.
78. Carrieri V, Madio L, Principe F. Vaccine hesitancy and (fake) news: Quasi-experimental evidence from Italy. Health Econ. 2019;28(11):1377-82.
79. Burki T. Vaccine misinformation and social media. The Lancet Digital Health. 2019;1(6):E258-E9.

For peer review only - http://bmjopen.bmj.com/site/about/guidelines.xhtml

80. Steffens MS, Dunn AG, Wiley KE, Leask J. How organisations promoting vaccination respond to misinformation on social media: a qualitative investigation. BMC Public Health. 2019;19(1):1348.

81. Daley MF, Narwaney KJ, Shoup JA, Wagner NM, Glanz JM. Addressing Parents' Vaccine Concerns: A Randomized Trial of a Social Media Intervention. Am J Prev Med. 2018;55(1):44-54.

82. Glanz JM, Wagner NM, Narwaney KJ, Kraus CR, Shoup JA, Xu S, et al. Web-based Social Media Intervention to Increase Vaccine Acceptance: A Randomized Controlled Trial. Pediatrics. 2017;140(6).

83. Penta MA, Baban A. Message Framing in Vaccine Communication: A Systematic Review of Published Literature. Health Commun. 2018;33(3):299-314.

84. Hendrix KS, Finnell SM, Zimet GD, Sturm LA, Lane KA, Downs SM. Vaccine message framing and parents' intent to immunize their infants for MMR. Pediatrics. 2014;134(3):e675-83.

For peer review only

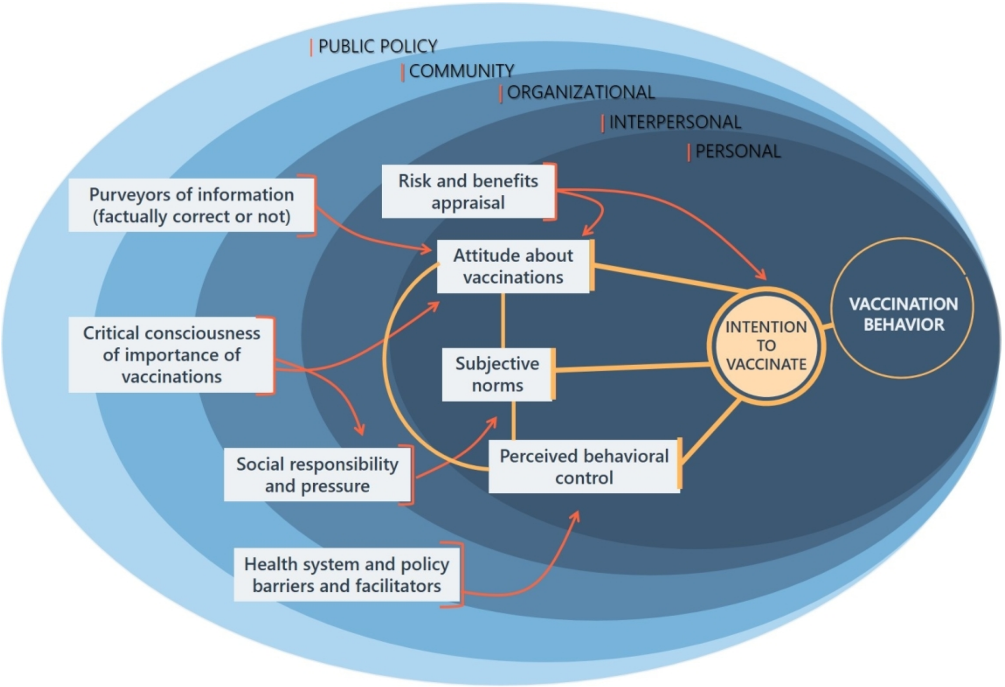

Figure 1. Preliminary conceptualization of different influences on vaccination behavior and VH to ground intervention development.

272x186mm (150 x 150 DPI)

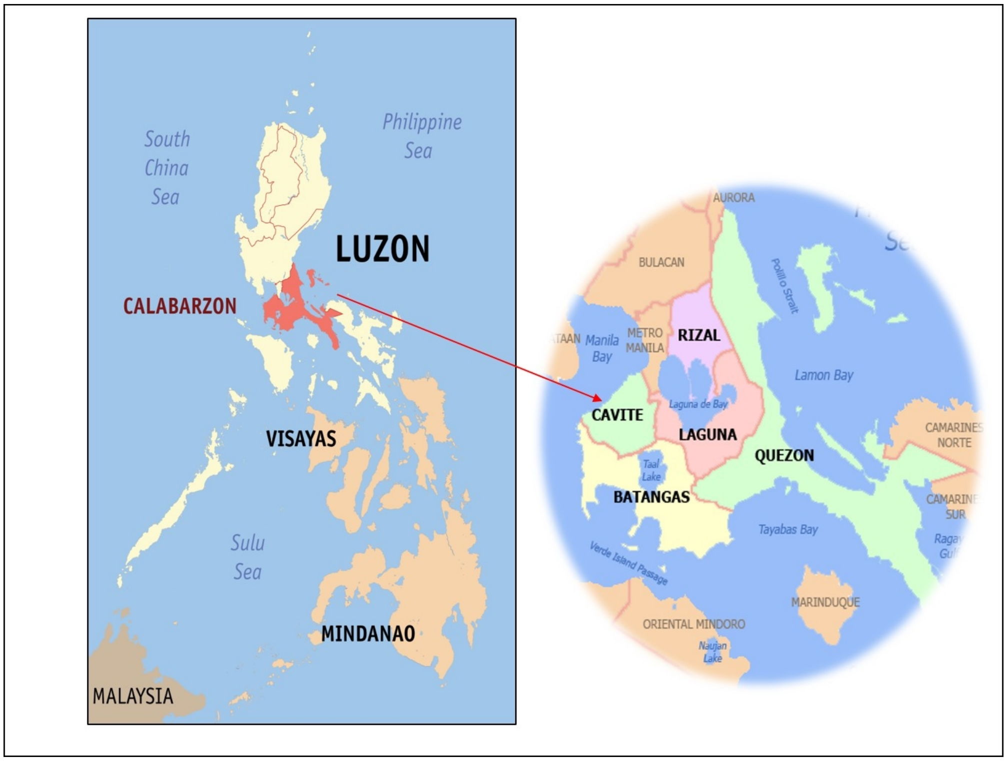

Figure 2. Location of Calabarzon Region included in the Project SALUBONG in the Philippines, 2020-2021. Map credits: a) Map of the Philippines showing the location of Calabarzon region; and, b) Political map of Calabarzon, courtesy of www.wikipedia.com (CC BY-SA3.0 and CC BY 2.5, respectively)

233x175mm (150 x 150 DPI)

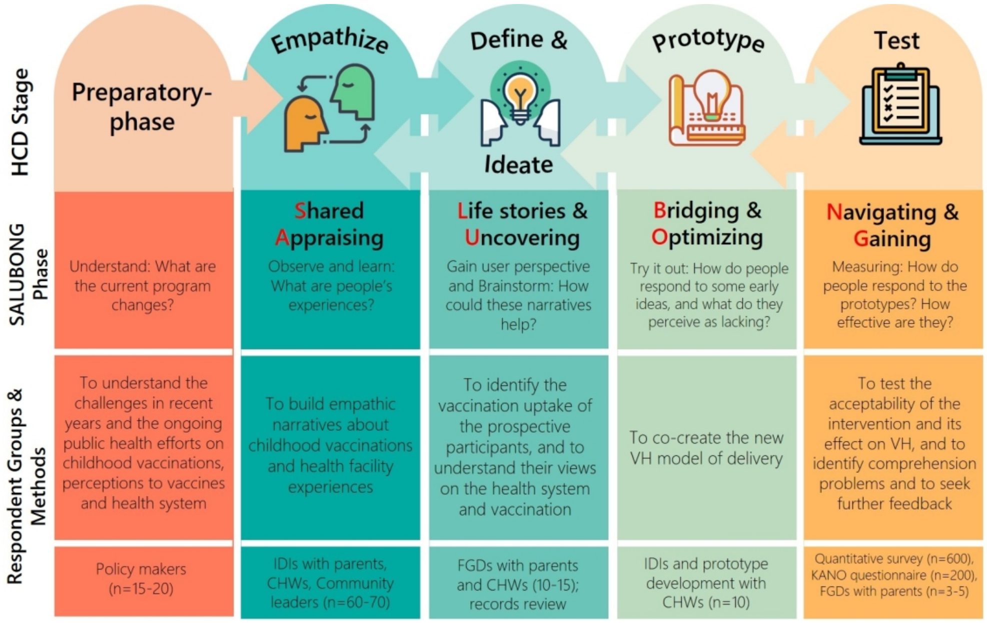

Figure 3. Summary of the Project SALUBONG guided by the human-centered design, 2020-2021. Icon credits: all icons made by Freepik (http://www.freepik.com) courtesy of www.flaticon.com.

285x176mm (150 x 150 DPI)

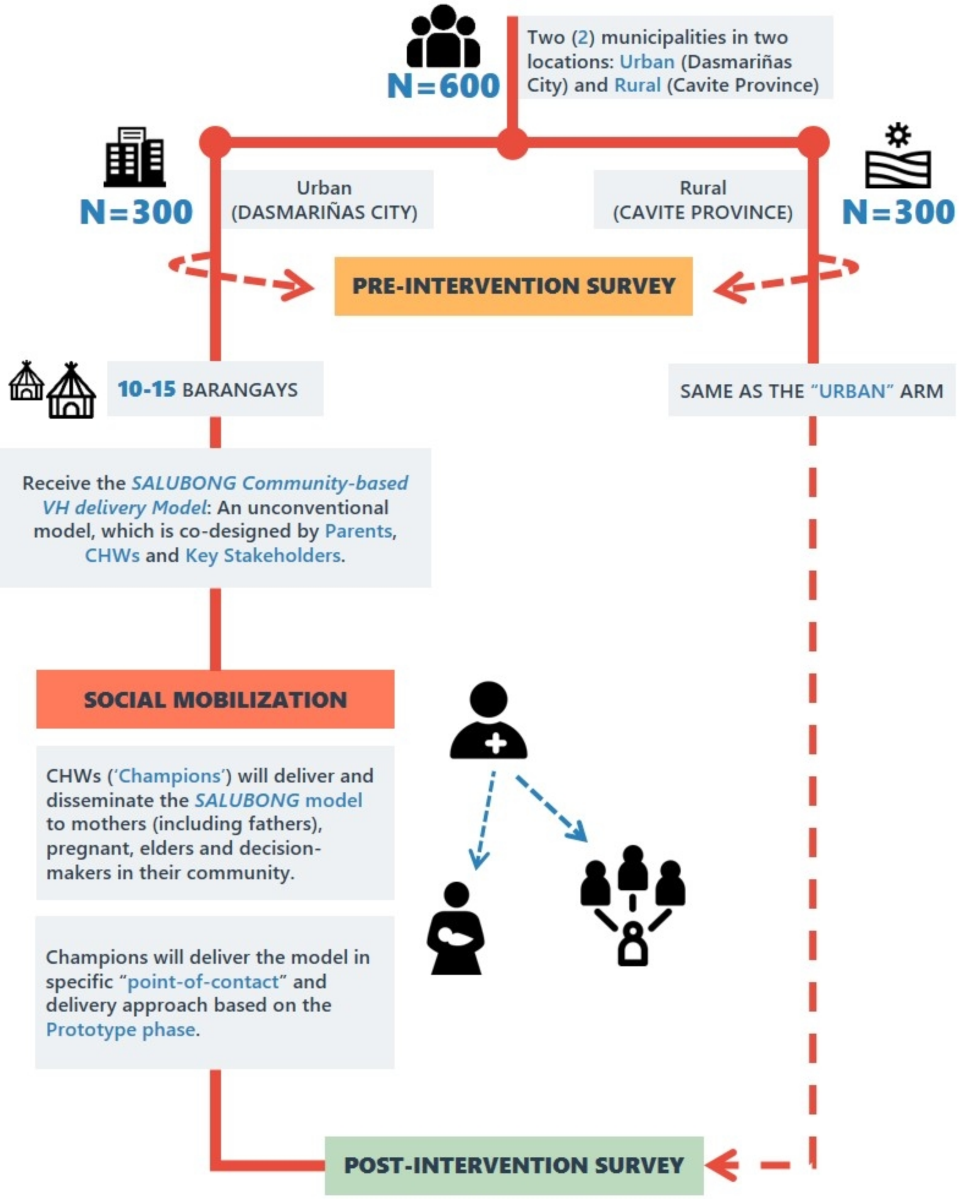

Figure 4. Study Design of Phase 4: "Navigating and Gaining". Icon credits: all icons made by Freepik (http://www.freepik.com) courtesy of www.flaticon.com.

135x165mm (150 x 150 DPI)

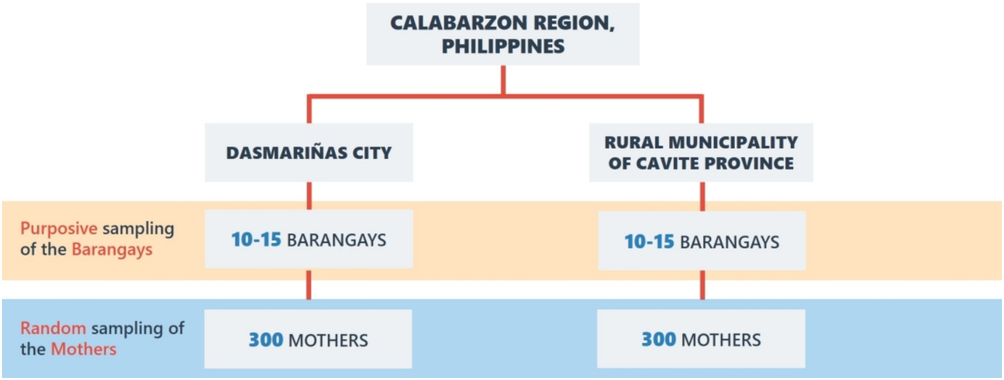

Figure 5. Schematic diagram of the sampling design of Phase 4.

323x121mm (150 x 150 DPI)

HEIDELBERG UNIVERSITY HOSPITAL

Heidelberg University Hospital | Im Neuenheimer Feld 672 | 69120 Heidelberg

06 November 2020

ADRIAN ALDCROFT
Editor, BMJ Open

Dear Mr. Aldcroft,

On behalf of my co-authors and I, please see enclosed our protocol paper entitled: "Project SALUBONG: Using human-centered design to build vaccine confidence via empathy and narratives in the Philippines" for your consideration in *BMJ Open*

Since the onset of a dengue vaccine controversy in late 2017, vaccine confidence has plummeted in the Philippines, leading to a measles and polio outbreak in early 2019. This protocol outlines a human-centered design approach to co-create and test an intervention that addresses vaccine hesitancy via narrative and empathy with and among families and health care workers.

We are confirming that this study is ongoing, and have not completed participant recruitment. Further, this paper is original, has not been submitted for review in other journals, and has been approved by all authors. We remain available to answer any questions you may have.

Yours sincerely,

SHANNON A. MCMAHON, PHD
Olympia Morata Assistant Professor at the HIGH, Heidelberg University
Associate at the School of Public Health, John Hopkins University

Im Neuenheimer Feld 130.3
69120 Heidelberg
Fon +49 6221 56- 36156
www.klinikum.uni-heidelberg.de

For peer review only

BMJ Open

How can Human-Centered Design build a story-based video intervention that addresses vaccine hesitancy and bolsters vaccine confidence in the Philippines? A mixed-methods protocol for project SALUBONG

Journal:	*BMJ Open*
Manuscript ID	bmjopen-2020-046814.R1
Article Type:	Protocol
Date Submitted by the Author:	12-May-2021
Complete List of Authors:	Reñosa, Mark Donald; Ruprecht Karls Universitat Heidelberg, Heidelberg Institute of Global Health; Research Institute for Tropical Medicine, Department of Epidemiology and Biostatistics<br>Wachinger, Jonas; Ruprecht Karls Universitat Heidelberg, Heidelberg Institute of Global Health<br>Bärnighausen, Kate; Ruprecht Karls Universitat Heidelberg, Heidelberg Institute of Global Health; University of the Witwatersrand, School of Public Health<br>Aligato, Mila; Research Institute for Tropical Medicine, Department of Epidemiology and Biostatistics<br>Landicho-Guevarra, Jhoys; Research Institute for Tropical Medicine, Department of Epidemiology and Biostatistics<br>Endoma, Vivienne; Research Institute for Tropical Medicine, Department of Epidemiology and Biostatistics<br>Landicho, Jeniffer; Research Institute for Tropical Medicine, Department of Epidemiology and Biostatistics<br>Bravo, Thea Andrea; Research Institute for Tropical Medicine, Department of Epidemiology and Biostatistics<br>Demonteverde, Maria Paz; Research Institute for Tropical Medicine, Department of Epidemiology and Biostatistics<br>Guevarra, Jerric Rhazel; Research Institute for Tropical Medicine, Department of Epidemiology and Biostatistics<br>de Claro, Nicanor III; Research Institute for Tropical Medicine, Department of Epidemiology and Biostatistics<br>Inobaya, Marianette; Research Institute for Tropical Medicine, Department of Epidemiology and Biostatistics<br>Adam, Maya; Stanford University School of Medicine<br>Chase, Rachel; Independent Researcher<br>McMahon, Shannon; Ruprecht Karls Universitat Heidelberg, Heidelberg Institute of Global Health; Johns Hopkins University Bloomberg School of Public Health
**Primary Subject Heading**:	Global health
Secondary Subject Heading:	Health services research, Health policy, Public health, Qualitative research, Patient-centred medicine

Keywords:	Protocols & guidelines < HEALTH SERVICES ADMINISTRATION & MANAGEMENT, Public health < INFECTIOUS DISEASES, HEALTH SERVICES ADMINISTRATION & MANAGEMENT, Health policy < HEALTH SERVICES ADMINISTRATION & MANAGEMENT, Health & safety < HEALTH SERVICES ADMINISTRATION & MANAGEMENT, Organisation of health services < HEALTH SERVICES ADMINISTRATION & MANAGEMENT

SCHOLARONE™
Manuscripts

I, the Submitting Author has the right to grant and does grant on behalf of all authors of the Work (as defined in the below author licence), an exclusive licence and/or a non-exclusive licence for contributions from authors who are: i) UK Crown employees; ii) where BMJ has agreed a CC-BY licence shall apply, and/or iii) in accordance with the terms applicable for US Federal Government officers or employees acting as part of their official duties; on a worldwide, perpetual, irrevocable, royalty-free basis to BMJ Publishing Group Ltd ("BMJ") its licensees and where the relevant Journal is co-owned by BMJ to the co-owners of the Journal, to publish the Work in this journal and any other BMJ products and to exploit all rights, as set out in our licence.

The Submitting Author accepts and understands that any supply made under these terms is made by BMJ to the Submitting Author unless you are acting as an employee on behalf of your employer or a postgraduate student of an affiliated institution which is paying any applicable article publishing charge ("APC") for Open Access articles. Where the Submitting Author wishes to make the Work available on an Open Access basis (and intends to pay the relevant APC), the terms of reuse of such Open Access shall be governed by a Creative Commons licence – details of these licences and which Creative Commons licence will apply to this Work are set out in our licence referred to above.

Other than as permitted in any relevant BMJ Author's Self Archiving Policies, I confirm this Work has not been accepted for publication elsewhere, is not being considered for publication elsewhere and does not duplicate material already published. I confirm all authors consent to publication of this Work and authorise the granting of this licence.

For peer review only

Title:

How can Human-Centered Design build a story-based video intervention that addresses vaccine hesitancy and bolsters vaccine confidence in the Philippines? A mixed-methods protocol for project SALUBONG

Name of Authors and Affiliations:

Mark Donald C. Reñosa[1,2], Jonas Wachinger[2], Kate Bärnighausen[2,3], Mila F. Aligato[1], Jhoys Landicho-Guevarra[1], Vivienne Endoma[1], Jeniffer Landicho[1], Thea Andrea Bravo[1], Maria Paz Demonteverde[1], Jerric Rhazel Guevarra[1], Nicanor de Claro III[1], Marianette T. Inobaya[1], Maya Adam[4], Rachel P. Chase[6], and Shannon A. McMahon[2,5]

[1]Department of Epidemiology and Biostatistics, Research Institute for Tropical Medicine – Department of Health, Muntinlupa, Philippines

[2]Heidelberg Institute of Global Health, Ruprecht-Karls-Universität Heidelberg, Heidelberg, Germany

[3]School of Public Health, University of the Witwatersrand, Johannesburg, South Africa

[4]Department of Pediatrics, Stanford University School of Medicine, Stanford, USA

[5]International Health Department, Johns Hopkins Bloomberg School of Public Health, Baltimore, USA

[6]Independent Researcher

Corresponding Author:

Shannon A. McMahon, Ruprecht-Karls-Universität, Heidelberg Institute of Global Health, University Clinic Heidelberg, INF 130.3, D 69120 Heidelberg, mcmahon@uni-heidelberg.de; Phone: +49 (0)6221 56-35049

For peer review only

ABSTRACT

Introduction: Since the onset of a dengue vaccine controversy in late 2017, vaccine confidence has plummeted in the Philippines, leading to measles and polio outbreaks in early 2019. This protocol outlines a human-centered design (HCD) approach to co-create and test an intervention that addresses vaccine hesitancy (VH) via narrative and empathy with and among families and health care workers.

Methods and Analysis: "Salubong" is a Filipino term that means to welcome someone back into one's life, reinforcing notions of family ties and friendships. We apply this sentiment to vaccines. Following the phases of HCD, guided by a theoretical framework, and drawing from locally-held understandings of faith and acceptance, we will conduct in-depth interviews (IDIs) and focus group discussions (FGDs) in rural and urban Filipino communities that witnessed dramatic increases in measles cases in recent years. During qualitative engagements with caretakers, providers, and policymakers, we will collect narratives about family and community perceptions of childhood vaccinations, public health systems, and opportunities to restore faith. IDIs and FGDs will continuously inform the development of (and delivery mechanisms for) story-based interventions. Once developed, we will test our co-created intervention(s) among 800 caretakers and administer a VH questionnaire prior to and immediately following the intervention encounter. We will use the feedback gained through the survey and Kano-style questionnaires to further refine the intervention. Considering the data collection challenges posed by the ongoing COVID-19 pandemic, we have developed workarounds to conduct data collection primarily online. We will use systematic online debriefings to facilitate comprehensive participation of the full research team.

Ethics and Dissemination: Ethical approval has been granted by the Institutional Review Board of the Research Institute for Tropical Medicine (No. 2019-44) and ethical commission of Heidelberg University, Faculty of Medicine (S-833/2019). Study findings will be disseminated in scientific conferences and published in peer-reviewed journals.

Strengths and Limitations of the Study:

- Project SALUBONG directly responds to calls in the literature for more community-based research on how VH can be addressed and how trust in the public health sector can be bolstered.
- Guided by families and communities, we will co-create an empathic intervention that places the health of children, the concerns of parents, and the needs of HCWs at the center to support trust in vaccines.
- Findings will inform future vaccine confidence efforts and contribute to broader policy discussions regarding VH in the Philippines and globally.
- Due to the COVID-19 pandemic, we have shifted some of our data collection online, and our study will be among the first in this setting to outline opportunities and pitfalls of remote qualitative research.

For peer review only - http://bmjopen.bmj.com/site/about/guidelines.xhtml

BACKGROUND

Vaccines are a cost-effective and safe way to prevent millions of deaths annually (1, 2). Although vaccines represent a seminal achievement in terms of mitigating disease, confidence in vaccines has decreased in many countries in recent years (3). This drop in confidence has contributed to stagnation or decreases in immunization rates, which in turn has resulted in outbreaks of previously controlled or domestically eliminated diseases such as measles and polio (4-7).

In 2019, the World Health Organization included vaccine hesitancy (VH) – the "delay in acceptance or refusal of vaccines despite availability of vaccination services" (8) – in its list of the top 10 global health threats requiring high-level attention and research (9). Literature that has sought to tease out the causes of VH emphasizes the '5Cs': complacency (regarding the severity of vaccine-preventable illness), constraints (psychological, financial or structural barriers), a lack of confidence (in vaccines and the broader health system), calculation (the degree to which individuals search for information about vaccines), and collective responsibility (a willingness to protect others) (10). More recently, scholars have started to consider the role of trust and unequal power dynamics in undermining vaccine uptake, describing how families have lost trust in the health system and feel that they have no voice in the face of state-mandated decisions or directives about vaccines (11-14).

Effective and efficient solutions to address VH are urgently needed, not only to mitigate the re-emergence of vaccine-preventable diseases (such as polio or measles (6, 15-17)), but also because the development and uptake of an effective vaccine is a cornerstone of controlling the ongoing COVID-19 pandemic (18, 19).

To date there is limited guidance in terms of how to successfully combat VH, most of which stems from high-income settings (20, 21). At the individual level, changing people's attitudes about vaccines has proven difficult, and successful interventions are limited (22). At the governance level, policymakers in high-income countries have considered or enacted laws to limit access rights and to punish those who reject vaccines (on non-medical grounds) by denying unvaccinated children admission to elementary schools and public playgrounds, and charging parents substantial fines (23). At the health system level, interventions involving medical professionals have considered how to broach the topic of vaccines in a non-judgmental but affirmative manner, how to listen to parents' vaccine decision-making (24), and how to facilitate vaccination directly through reminders, prompts, or by reducing logistical barriers (22).

More recent studies also highlight opportunities in terms of video-based vaccine promotion (24) and educational messages in the form of graphic pictures and anecdotes (focusing on the consequences of not getting a child vaccinated) (25). Several studies have employed Human-Centered Design (HCD), an approach to co-develop interventions with end-users (26). This methodology has led to the creation of mobile apps, education materials, provider guidelines, and the re-design of a health facility, all in the interest of bolstering vaccine uptake (24, 27-31). Results of these HCD studies suggest that the approach supports stronger patient and community engagement (24, 27, 30).

For peer review only - http://bmjopen.bmj.com/site/about/guidelines.xhtml

Relatively little attention has been paid to VH and ways to address VH in low- and middle-income countries (LMICs) (14, 21, 24, 25). This is particularly problematic for at least three reasons: 1) a majority of the world's vaccine-preventable deaths occur in LMICs (32); 2) public health and immunization structures in LMICs are insufficiently equipped to address VH while rolling out national immunization campaigns and fielding other child health challenges; and 3) in the event of an outbreak of a vaccine-preventable disease, survival rates and containment possibilities are markedly reduced in LMICs where poor structural conditions and extreme poverty can exacerbate pre-existing vulnerabilities (33-35).

The Philippines, an archipelago with a population of more than 105 million, is among the LMICs that are currently experiencing an unprecedented erosion of public trust in childhood vaccinations (36-38). Vaccine confidence fell from 93% of adults "strongly agreeing" to the importance of vaccination in 2015 to 32% in 2018 (36). This is reflected in measles vaccination rates of children under-five, which fell from 88% in 2014 to 55% in 2018 (39). Results from a small 2019 study conducted in two urban communities in Manila, the capital of the Philippines, found that 36% of responding parents had hesitated to give at least one vaccine and/or refused at least one vaccine for their children (40). These sharp declines are associated with a dengue vaccine controversy in 2017 and the ensuing misinformation that eroded faith in vaccine safety (36-38, 41). This erosion led to the country losing its 19-year polio-free status, and sparked measles outbreaks across several islands in 2019, with 47,871 cases including 632 deaths (as compared to 2,789 cases and 25 reported deaths in 2018) (39, 41, 42). The Department of Health (DOH) of the Philippines has therefore made it a priority to win back the trust and confidence of the public in vaccination (43). The DOH - in partnership with the World Health Organization and UNICEF - has strengthened routine immunization via the launch of door-to-door immunization campaigns to increase vaccine uptake and to reach unvaccinated children (16, 44). Although recent data has indicated signs of possible recovery, with the proportion of people agreeing to the importance of vaccines increasing, gains in terms of perceived vaccine safety and effectiveness are less substantial (3). More tailored and innovative initiatives are necessary to regain community trust in vaccines.

The SALUBONG Intervention

"Salubong", the moniker for this research, is a Filipino term that means to welcome someone into one's home or life. In the Philippines, the largest predominantly Christian country in Asia, the term Salubong describes a Catholic dramatization of the resurrected Jesus encountering his mother Mary, which is a central part of the Easter week across the Philippines. This encounter, which is not liturgically accounted for, culminates in Jesus – if briefly – reuniting with his mother (45). From this, Salubong has developed into a Filipino tradition that celebrates the beauty of re-connecting with important figures from one's past. The Salubong tradition is commonly observed at international airports throughout the Philippines, where many Filipinos are waiting for their loved ones to return home after having sought employment

For peer review only - http://bmjopen.bmj.com/site/about/guidelines.xhtml

abroad (the diaspora has fostered an undertone of longing and anticipation in many Filipino homes) (46, 47). The homecoming of a loved one is viewed as a special and festive event accompanied with a warm embrace and sumptuous meal.

In using this term as our project moniker, we aim to signal to communities that unwelcoming vaccines into homes or lives is a current dilemma, like many of life's dilemmas, but in unwelcoming there is a progression to re-welcoming. Salubong is intertwined with notions of acceptance, compassion and understanding, and sends the message that we will not rely on an intervention that uses the blunt tool of scientific reason or which excludes population perceptions (particularly as this has proven ineffective in relation to VH (25)). Instead, we will employ HCD (48) to co-develop an intervention together with wary families and communities to encourage them to reconsider their views on childhood vaccinations.

Our central thesis is that by drawing from local narratives, designing, refining, and ultimately testing a story-based intervention that bridges caretakers (e.g., parents, other family members, legal guardians), policymakers, health care workers (HCWs), community leaders, and community health workers (following the terminology in the Philippines, where small administrative communities are termed *barangays*, termed 'barangay health workers' (BHWs) in the context of this study), we will lay the foundation to build a meaningful campaign that revives faith in vaccines. This foundation will contribute to the sustainable prevention of outbreaks of vaccine-preventable diseases.

Study Objective

The purpose of this study is to understand local perspectives of VH in the Philippines, and to develop and pilot a health promotion intervention to address VH. Following the multiple stages of HCD, we will co-design, develop, and iterate a community-based intervention (26). The developed intervention will then be piloted to assess effectiveness and acceptability.

Sub-objectives include:

1. To describe caretakers, policymakers, and community perceptions of the public health system and vaccines.
2. To gather narratives regarding childhood vaccination and health facility experiences from caretakers, HCWs, BHWs, and community leaders (including real-life dialogue between families who delay and refuse vaccinations).
3. To design, pilot, refine, and assess the immediate impact of a picture or video-based intervention with caretakers and community stakeholders.

METHODS AND DESIGN

Theoretical underpinning

We draw on the Theory of Planned Behavior (TPB) (49) to acknowledge how control beliefs (whether to vaccinate and the consequences of this decision), attitudes about vaccinations, and normative beliefs

For peer review only - http://bmjopen.bmj.com/site/about/guidelines.xhtml

(i.e. notions of social responsibility; subjective norms influenced by social or cultural aspects) shape the intention to vaccinate and vaccination uptake. Such normative beliefs are of particular relevance in this context, as vaccination uptake has a distinct social responsibility dimension. High vaccination coverage is required to protect those who cannot (yet) get vaccinated, but the possibility of 'freeloading' exists for those who refuse vaccinations due to the risks for the individual, yet can still benefit from others being vaccinated.

A number of studies have employed the TPB to explain VH, supporting its utility and describing potential starting points for interventions (50-52). A recent meta-analysis suggests that the TPB can explain over 50% of the variance in intention to vaccinate, with attitudes and normative beliefs being stronger predictors than perceived behavioral control (53). A 2017 systematic review of trialed vaccine confidence interventions found few interventions with limited efficacy that aimed at changing individuals' attitudes or their awareness of social norms, and instead identified interventions aimed at reducing barriers and therefore increasing behavioral control as a more promising pathway (22).

At the core of the current VH crisis in the Philippines is a recent vaccination controversy, which heavily affected the trust in HCWs, the health system, and other vaccination stakeholders (38). We therefore are also informed by the Social Ecological Model (SEM) to understand how vaccination attitudes and behaviors are shaped on individual, interpersonal, organizational, community and public policy levels (54). Kumar and colleagues identified factors across all socio-ecological levels that affect influenza vaccine uptake (55), and a number of authors have argued for including multiple socio-ecological levels when considering how to address VH (56, 57). We will draw on the SEM to gain a better understanding of how stakeholders on different levels perceive barriers or facilitators for vaccinations, and how systematic changes or awareness can increase uptake.

Based on these theoretical models, we developed a framework which guides this project (**see Figure 1**). With the objective to increase individual caretakers' intention to vaccinate their child, we aim at empathetically addressing their attitudes, norms, and perceived behavioral control on an individual level (i.e., the caretaker receiving the intervention). In developing this intervention, we however acknowledge that factors such as organizational barriers, purveyors of information, or social pressures influence vaccination perception and uptake across several levels of the SEM.

Study setting

The Republic of the Philippines consists of more than 7,000 islands divided into 17 administrative regions (58). This study will be conducted in the Calabarzon region, which has an estimated population of 14 million (with approximately 80% being of Roman Catholic faith, and the remaining 20% predominantly belonging to Christian and Muslim denominations), including 1.2 million children under-five (**see Figure 2**) (59). Calabarzon experienced a 300% increase in measles cases in 2019 as compared to 2018 (39, 60, 61). The region is middle-income and predominantly consists of agriculture, fishing, manufacturing and high technology industries (62, 63). Calabarzon is composed of five

For peer review only - http://bmjopen.bmj.com/site/about/guidelines.xhtml

provinces, 20 cities, 123 municipalities and 4,018 *barangays* ("small communities"). The project will be conducted in Dasmariñas City and rural municipalities of Cavite province, which were purposively selected to reflect both rural and urbanized conditions, and to capture varied socio-demographic factors and health facility related experiences in terms of child health and vaccinations.

Study Population

Our study population will include community leaders (such as the *barangay* captains and councilors on health), caretakers of young children, policymakers, HCWs (municipal/city health officers, nurses, and midwives), and BHWs (community-based health volunteers who help and assist nurses and midwives in the delivery of essential health care programs (64)). Ethnicity, race, political orientation, religion and class are not criteria for inclusion or exclusion in this study. Caretakers, HCWs, BHWs and community leaders will be eligible to participate in the study if they live within Cavite Province and Dasmariñas City. Participants must be at least 18-years-old or an emancipated minor (who are 15-17 years old but with children under-five) to participate. Incapacitated persons are excluded.

Study Design

Informed by HCD (26), this study employs a mixed methods, exploratory sequential design, drawing on qualitative and then quantitative methods (65). We will first use qualitative methods (IDIs and FGDs) and then quantitative methods (pre-post surveys and Kano questionnaires). **Figure 3** shows the summary of the five study phases (a preparatory phase followed by the four phases of HCD), along with specific objectives and expected corresponding outputs. Within any given phase, iteration and repetition will typically be necessary. The ultimate goal of the process is to ensure that the fundamental design of a product or program reflects what users want and works in the setting where they will be using it. Data collection for this study will occur from September 2020 through August 2021.

Sample Sizes and Sampling Technique

We will partner with HCWs and BHWs who keep lists of caretakers of young children to identify eligible households, particularly families with various experiences or perspectives on vaccines in order to help us capture a range of insights in terms of vaccine attitudes and behaviors.

Qualitative component: Purposive sampling will be utilized for the qualitative components of the Pre-phase and Phases 1-3 to gain a maximum range of perspectives and depth of information; sample size estimates are guided by saturation estimates and outlined in **Figure 3** (66, 67). To select the study sites, initially, all municipalities of Cavite province and Districts in Dasmariñas City will be listed. We will select one municipality in Cavite Province and one district in Dasmariñas City with the lowest Expanded Program on Immunization coverage for the period of 2018-2019; this approach maximizes the probability of finding caretakers who delay or refuse childhood vaccinations. For each selected

For peer review only - http://bmjopen.bmj.com/site/about/guidelines.xhtml

municipality and district, one to two *barangays* with the highest number of children under-five will then be purposively selected.

Quantitative component: During Phase 4 we will rely on household survey data. The estimated sample size per group (intervention and control arms in both rural and urban areas) is 200, which will result in a total of 800 survey responses sought. This will allow us to detect a difference of 15% in the binary outcome between intervention and control groups in each area with an 85% response rate, 5% type I error rate, and 20% type II error rate. To select the study sites, a multi-stage stratified random sampling frame will be used. The sampling scheme is illustrated in **Figure 4**.

The four *barangays* (two from Dasmariñas City and two from a rural municipality in Cavite) will be purposively selected based on the number of annual births in the most recent report available (preferably 2018-2019) in the Rural Health Unit/City Health Office. We will select the two *barangays* with the highest number of births in their respective region and randomly assign study arms.

In each of the selected *barangays*, a list of caretakers with children under-five will be obtained from the midwife or the BHWs. All caretakers listed will be invited to participate in the study. In the event that the number of interviewed caretakers does not reach the envisioned sample size of 200 per area and study arm, we will continue sampling in the *barangay* with the next most births in the area in the matter described above.

Data Collection Preparation and Data Collector Training

Before the onset of data collection, we will communicate with Department of Health (DOH) officials (national, regional and provincial offices) to explain study objectives and procedures, and discuss and resolve any concerns regarding data collection. We will also seek their permission and support to conduct the project in the selected primary health care facilities. Official communication explaining the nature, study objectives, and procedures will be sent to local government executives and community leaders. Spot maps of the community, if available, will be requested from the Rural Health Units or community health centers. The spot maps will be used to locate prospective families in communities. Courtesy calls to local officials and leaders will be made to seek their support to conduct the project in their respective localities. All required formal endorsements (i.e., memorandums of agreement) will have been granted prior to data collection.

The research team will conduct IDIs, FGDs, and quantitative surveys with the assistance of field interviewers in either English or Filipino, depending on the preference of the participant. Interviewers will be fluent in English and Filipino with bachelor's level education in nursing, midwifery or social sciences. Interviewers will be trained for five days to collect the data with instruments and online platforms for data collection for this study. Training topics will include modules on VH, interviewing and/or surveying techniques, research ethics, software resources, Kano analysis and qualitative and quantitative methods.

For peer review only - http://bmjopen.bmj.com/site/about/guidelines.xhtml

Data Collection

Data collection will be conducted in five phases: One preparatory phase to develop an understanding for the current situation with regards to vaccination policies and challenges, including but not limited to the ongoing COVID-19 pandemic, and the four phases of HCD.

Preparatory Phase

We will conduct IDIs with policymakers (n=15-20) to understand the current challenges for ongoing public health efforts on childhood vaccinations, perceptions of vaccines, and the health system. We will also explore how the current COVID-19 pandemic poses challenges and opportunities to health education and vaccination efforts.

Phase 1. Shared Appraising (EMPATHIZE PHASE):

This phase of HCD aims at gathering information about how users frame a childhood vaccination problem, how they situate themselves in relation to the problem (probing on socio-cultural context) and learning which factors would motivate them to address the problem.

IDIs with caretakers of children under-five: We will conduct IDIs (n=60) with caretakers of children under-five. In each selected *barangay* in Cavite province and Dasmariñas city, we will purposively select 30 caretakers recommended by a nurse or midwife who will have reviewed vaccination records in order to identify families that have delayed or refused childhood vaccines. Based on these IDIs, we will build holistic narratives about family perceptions of the public health system and encounters with childhood vaccinations. Participants with particular vaccination or VH experiences will be asked if they are willing to be video-recorded as they tell their personal story related to vaccines. These video-recorded interviews will aid pre-development of the intervention.

IDIs with HCWs and community leaders: IDIs (n=10) will also be conducted among HCWs (municipal/city health officers, nurses, midwives) and community leaders (specifically the *barangay* captain and councilors for health) of the selected *barangays* to describe their experiences related to childhood vaccination in their respective community or health facility.

Refinement of intervention storyboards: Following each interview, the preliminary storyboards developed as a result of the initial IDIs (i.e., 10-15 IDIs) will be presented to participants. Participants (caretakers, HCWs) will be asked to conduct a think-aloud exercise while flipping through these storyboards. After each 10-15 IDIs, or at the end of each week of data collection, the storyboards will be edited and refined based on participants' comments. These revised storyboards will be included in the following round of IDIs for further refinement.

Phase 2. Life stories and Uncovering (DEFINE & IDEATE PHASE):

In this phase, end-users will suggest ideas to address the problem in collaboration with a research team.

For peer review only - http://bmjopen.bmj.com/site/about/guidelines.xhtml

Records Review: From the selected *barangays*, the research team will seek the help of BHWs to identify potential participants based on childhood vaccination records. We will purposively select 50 potential participants in each selected *barangay* of Cavite province and Dasmariñas City and review their vaccination record through Target Client Lists and Individual Treatment Records. Each case will be allocated to one of three categories:

1. *Fully Immunized Child:* Children who complete one dose of BCG, three doses of Oral Polio Vaccine (OPV), three doses of Diptheria-Pertussis-Tetanus (DPT), three doses of Hepatitis B and one dose of Measles Vaccine BEFORE a child's first birthday. These are the children who received their vaccinations within the National Immunization Program schedule.
2. *Completely Immunized Child:* Children who receive one dose of BCG, three doses of OPV, three doses of DPT, three doses of Hepatitis B and one dose of Measles Vaccine AFTER a child's first birthday. These are children who have delays in receiving vaccination based on the recommended National Immunization Program schedule.
3. *Refusal to vaccinate:* Children with the remark "refused" in the Target Client Lists will be cross-validated with the respective Individual Treatment Records to further review their reasons of refusal.

This process will allow us to initially stratify the participants for FGDs based on their children's vaccination status, preventing contamination and conflict during discussions. Additionally, we will use the records review to confirm delays in children's vaccination schedules, even if these children were later fully immunized.

Focus Group Discussions: We will conduct FGDs (n=10-15) with caretakers, stratified (based on the records review and validation of child vaccination cards) into three groups: 1) in favor, 2) delay and 3) refusal of vaccination, to understand how these views evolved or persisted. We will explore the socially held attitudes toward the public health system and vaccines. We will also conduct unstratified FGDs among BHWs of the sampled *barangays* to understand their community and health facility experiences with childhood vaccinations. Results of these FGDs will inform the development of storyboard flip-boards that will be refined in the next phases of the project. To ensure that the design itself of the intervention resonates with the intricate details of Filipino demographic and cultural dynamisms, we will collaborate with local animators with years of groundwork experience in developing interventions in the Philippines who will accompany FGDs and graphically record ideas and concepts in real-time, which can be discussed among participants.

Phase 3. Bridging and Optimizing (PROTOTYPE PHASE):

In this phase, prototypes and products will be developed and tested in real-world settings with actual users via actual delivery systems.

IDIs with HCWs and BHWs: The draft intervention, together with data from our FGDs and IDIs will be presented to HCWs and BHWs. We will conduct 10 IDIs with HCWs and BHWs to get their

For peer review only - http://bmjopen.bmj.com/site/about/guidelines.xhtml

perspectives and recommendations regarding additional information that needs to be included or refined, namely related to how, when, and by whom the intervention should be delivered. These IDIs will guide the research team in terms of preferred medium (e.g. paper versus video-based presentation) and favorable delivery approach (e.g. spoken text to accompany the intervention, one-on-one vs. group delivery, a stand-alone activity vs. nested in existing outreach, etc.). The result of this phase will allow us to further refine and finalize the intervention, and to determine the "point of contact" for the delivery of the intervention in Phase 4.

Phase 4. Navigating and Gaining (TESTING PHASE):

In this phase, the intervention will be introduced to and tested with a larger sample, and large-scale feedback will be sought.

Pilot Testing: We will test the developed intervention versus a control intervention (a standard health education, unrelated to vaccines or VH) in four *barangays* (divided into urban and rural areas) in the same municipality and district selected in the previous phases. **Figure 4** shows the detailed design of the pilot phase. Before and after the delivery of the intervention, the research team will administer short surveys. The pre- and post-intervention surveys (based on best practices for measuring VH (8, 68)) will quantify participants' knowledge, attitudes, and practices regarding vaccination and VH, and the post-intervention survey will additionally assess any changes in knowledge or attitude. We will also collect participants' socio-demographic characteristics (i.e., *barangay* of residence, caretakers' age, sex, civil status, occupation, number of children and education level) and vaccination status of children to allow for further analyses and the identification of potential sampling biases. We will store contact data with an intention to reach out to families in future intervals to determine whether vaccination completion rates differed between intervention and control groups. We will revise the survey tool based on outcomes from phases one and three.

Intervention Feedback: After pilot testing, a subset of 200 participants (100 participants randomly sampled from each the urban and rural intervention arms) will be asked a short series of closed-ended questions about features of the intervention and feedback on how the intervention made the user feel based on Kano analysis methodology (attractive vs. essential) (69). After the Kano survey, 10-15 participants in each area (n=50) will be purposively selected based on critical case sampling and invited to IDIs to identify comprehension problems and to seek further feedback in smaller groups. Since this study only aims to pilot test the developed intervention, the feedback on the intervention will be useful for further refinement before future trials.

Patient and Public Involvement

Neither clients nor the public were directly involved in the design, recruitment, or conduct of the study; their only involvement is as research participants. However, following the tenets of HCD, the intervention is co-developed with research participants over the entire course of the study. Participants

For peer review only - http://bmjopen.bmj.com/site/about/guidelines.xhtml

are not involved in initial recruitment, but those participants completing an IDI may be asked to provide contact details of others who might be interested to participate in the study. Participants are not involved in data analysis or the dissemination of findings. The final results will be shared with policymakers, health programmers, and other stakeholders via roundtable discussion, and in the form of articles published in peer-reviewed journals or policy briefs. Research participants will be given access to the final intervention and be informed through their BHWs and other channels embedded in the community, and have the option to contact study staff for a detailed description of study findings at any time.

Data Collection in times of COVID-19

All IDIs and FGDs were initially planned to be conducted in-person. However, the ongoing pandemic and measures taken to curb infection rates pose unique challenges to conducting research. Considering the time-sensitive nature of this project, we have developed new operating procedures for conducting online data collection, to ensure minimal risk for participants and the research team concerning COVID-19, and in compliance with the recommendations by the European Medicines Agency (70) and the Philippines Inter-Agency Task Force (IATF) (71). This includes informed consent processes and the engagement of research team members who are unable to travel to the study site. Until the situation changes, only strictly necessary visits will be performed at sites.

Remote recruitment and consenting: We will contact prospective participants via email or a phone call to briefly introduce the study aims and invite them to participate. After this initial phone call or email, we will set an appointment for the comprehensive discussion of the study and to answer questions. An information sheet (including study aims, procedures, and expected risks and benefits) and consent forms will be sent to potential participants in advance via email or courier. If participants agree to enroll in the study, we will ask them to sign the consent form during a recorded Skype or Zoom video call, and to take a picture while holding the signed consent form. The signed consent forms will be returned to the research team as scanned or photographed copy, or per courier (as preferred by the participant; courier costs will be covered by the research team). Participants will receive duplicate copies with the signature of the interviewer per courier.

Focus Group Discussions: At the time of writing (October 2020), we continue to hope that by the beginning of 2021, when we plan to conduct FGDs, small group discussions in person with appropriate distance will be possible. However, we will conduct remote FGDs if necessary to ensure the safety of participants and research team. In this case, we will draw on the assistance of local stakeholders (HCWs, BHWs etc.) to orient participants on group discussions via a web-based platform.

Intervention pilot-testing: If necessary, amid the pandemic and associated lockdowns, the intervention (VH and control) and surveys (pre and post) will not be delivered in person but instead via an online platform of the participant's choosing. During the presentation of the intervention, there will be no interaction between the researchers and participants to prevent any sort of biases or contamination of the results. We will similarly administer the quantitative survey via an online platform. All online

For peer review only - http://bmjopen.bmj.com/site/about/guidelines.xhtml

processes will be pilot tested prior to the implementation to assess feasibility and operational challenges. If feasible, the survey will be in a self-administered format (i.e., interviewers will send the survey link and participants will input their answers in the online form). However, if deemed not feasible, the research team will switch to an interviewer-assisted survey format (i.e., interviewers will read the questions to the participants and are responsible in inputting the answers in an online form).

Systematic Debriefings: As a number of research team members will not be able to travel to the study site due to COVID-19 travel restrictions, we will employ remote systematic debriefings (72) to discuss and triangulate findings, to amend interview guides and to refine lines of inquiry. The weekly debriefings will also allow us to continuously assess, discuss, and refine study tools, data collection procedures, and emerging issues in data collection as a means to ensure fidelity to the tenets of high-quality interviewing. Additionally, in light of the timely relevance of vaccination research in the context of the ongoing pandemic, we will use these debriefings to discuss emerging topics and potential probing approaches with regards to adult and COVID-19 vaccination and continuously refine data collection tools accordingly.

DATA PROTECTION AND ANALYSIS

Data quality checks and cleaning process

Most of the data in the project will be primary and collected by the research team. Interviews will be audio-recorded using digital or online audio-recorders. All recorded information will be transcribed verbatim and translated into English. For all qualitative data, the research team collecting and analyzing the qualitative data will be directly responsible for quality checks of audio recordings, interview notes and qualitative transcripts. Random checks of transcripts will be conducted to ensure quality.

All quantitative data will be entered into a customized Microsoft Access data entry system with a built-in validation program. The data management unit (DMU) of the Research Institute for Tropical Medicine's Department of Epidemiology and Biostatistics will be responsible for quality checks. DMU staff will reach out to the SALUBONG team in the event of questions or data inconsistencies.

Data storage and protection

All data collected by the research team will be stored according to Germany and Philippines regulations. At the point of data collection, unique codes will be assigned to all participants. A master sheet linking the code with identifying information (including a name and, when feasible, contact information) will be kept in a secure (locked/password-protected) location that is accessible to the investigators. Only the research coordinator will have access to and manage this master sheet. The master sheet will facilitate the process of revoking information should a participant decide (at a later point) that they wish to have their data removed from the study. Further, audiotaped interviews will also be stored in locked/password-protected computers controlled by the research coordinator. All data will be accessible

For peer review only - http://bmjopen.bmj.com/site/about/guidelines.xhtml

only to those within the research team, but can be made available to others upon reasonable request after approval from ethical review boards.

Data analysis

While the work is divided into phases, the ultimate aim of this study is to analyze and apply findings continuously across phases to address the overall study objective. The Principal Investigators and Co-Investigators will be directly responsible for the triangulation of findings across data sources and the development of an integrated interpretative analysis.

Qualitative analysis: The qualitative component of this study will be analyzed based on constructivist grounded theory as outlined by Charmaz (73). The application of this approach will help us generate theories on why caretakers would or would not agree to childhood vaccinations, and it will allow us to investigate the constructions and underlying processes of caretakers' perceptions, so that informed and relevant approaches can be applied to help resolve highly salient concerns. NVivo Pro 12 (QSR International Pty Ltd. Version 12, 2018) will support qualitative analysis.

Quantitative Analysis: The quantitative component of this study will be analyzed using descriptive and inferential approaches. Earlier phases of the study will identify relevant dimensions of knowledge and attitudes to assess in the survey; at its most basic, the analysis will assess binary improvement (1) versus no improvement (0) across those dimensions comparing after and before intervention exposure. Additional analyses will describe and compare the rates of generally favorable versus unfavorable attitudes toward vaccination. Exploratory analyses will seek to uncover sociodemographic variables related to pre-intervention response patterns as well as post-intervention change patterns.

Kano-style questions place user ratings of product or service features on a two-dimensional scale (69). One dimension is satisfaction, which can range from frustration to delight. The other is functionality, which ranges from not functional to highly effective (69). Hence, Kano analysis of post-intervention survey responses to Kano-style questions will be used to prioritize resource allocation in improving the intervention functionality, with the intention of causally affecting customers' satisfaction. The results will then be used for the revision or re-development of intervention features for future, large-scale testing.

Ethics and Dissemination

Ethical approval has been granted by the Institutional Review Board of the Research Institute for Tropical Medicine (No. 2019-44) and the ethical commission of Heidelberg University, Faculty of Medicine (S-833/2019), both recognized Ethical Review Committees. The investigators will consistently respect the principles of ethical research on human subjects described in the Declaration of Helsinki. Written informed consent will be obtained from all eligible participants before data collection begins and after the participants have been fully informed about the study. Study findings will be disseminated in scientific conferences and published in peer-reviewed journals, as well as via policy

For peer review only - http://bmjopen.bmj.com/site/about/guidelines.xhtml

briefs and roundtable discussions. We will adhere to specific reporting guidelines for all publications as applicable, such as the Standards for Reporting Qualitative Research (SRQR) or the Consolidated Criteria for Reporting Qualitative Studies (COREQ).

DISCUSSION

Although vaccination uptake and VH are central parts of the current public health and global health discourses, few interventions have proven efficacious in sustainably increasing vaccine confidence. In this study protocol, we explain how we will work with clients, providers, and policymakers to develop a narrative-based intervention to increase vaccine confidence in the Philippines. Guided by the tenets of HCD we will co-develop and iterate this intervention to address the needs and expectations of those it is developed to support.

HCD is a promising new approach in the development of global health interventions (48). In our application of this methodology we will move beyond the way it currently is often applied and extend the understanding of 'clients' (following terminology from HCD field understood here as the end-users of the intervention) to those involved across several levels including: caretakers who are deciding whether to vaccinate their children (clients in the sense of 'target population'), HCWS and BHWs who interact with caretakers (clients in the sense of those who will – or will not – incorporate the intervention into their service delivery), and policymakers and key vaccination stakeholders whose decisions and policies shape the vaccination landscape (clients in the sense of those who decide the implementation of large scale interventions).

We also expand the existing body of literature on HCD-based vaccine confidence interventions by drawing on a theoretical framework to inform our sampling and design procedures and using well-established behavior change theories to guide data collection activities. This for example includes identifying topics to be probed for in IDIs (e.g., perceived behavioral control about getting a child vaccinated), but also those topics with a clearly social dimension to be focused on in FGDs (e.g., social responsibility of vaccinations). We therefore do not employ a purely inductive approach for conducting qualitative research, as is common in global health qualitative research and HCD approaches, but allow for the existing literature and established theories to guide our approach.

Previous studies demonstrate that decision-making surrounding vaccine is complex, and that those who provide vaccine-related information are in a unique position to sway or break vaccine acceptance (74, 75). A high general trust in the health authorities and providers that decide what vaccines to introduce is a deciding factor in vaccine uptake (76). However, trust and source credibility play a pivotal role not only in direct communication between healthcare providers and their clients, but also across all levels: Health-related controversies have been shown to disrupt the trust healthcare providers have in political agents, or implementing government institutions have in those who make the respective policies (38). Our inclusion of stakeholders from all levels in the design process will support our ability to identify pathways to rebuild both confidence in vaccines and trust in the general system.

For peer review only - http://bmjopen.bmj.com/site/about/guidelines.xhtml

The Dengvaxia controversy in the Philippines highlights the pace and reach of misinformation regarding the effectiveness and safety of a vaccine, causing a drop in general vaccine confidence and rolling back decades of immunization work (36, 38). Dayrit and colleagues (38) stated that the communication gaps among the health authorities themselves, as well as a lack of transparency, proliferation of non-scientific speakers in the mainstream media, and distorted messages in social media played a key role in the panic felt among Filipino families. The Dengvaxia controversy was exacerbated by an exceptionally rapid spread of (mis)information via social media channels (36, 38). In the broader context of vaccination, factually false or misleading information shared in online forums or via social media, often in the form of short quotes or emotionalized images, is considered a central contributor to anti-vaccination narratives (77). In many cases, countering these narratives is challenging as it requires longer and complex explanations of vaccination risks and benefits, which are not as easily spread through social media (78, 79). We are taking this into consideration while developing our narrative-based intervention in part or entirely in a form that can be easily relayed (e.g., as a short video or even a GIF). With vaccine confidence or uptake interventions delivered via social media showing promising results (80, 81), we explicitly include this notion in our HCD-based discussion of promising delivery strategies.

The Dengvaxia controversy has been repeatedly linked to the steep decline in vaccination rates and vaccine confidence in the Philippines (3, 36). However, to the best of our knowledge, there so far has not been any qualitative exploration of how this case has shaped narratives about vaccines, health programming and the health system in general. Furthermore, there has been remarkably little research highlighting Filipino perspectives on how to move forward after the controversy. The SALUBONG project will thus provide robust, culturally-attuned data that can inform future programs and policies to rebuild trust and confidence in relation to vaccines (22, 82, 83).

Finally, similar to many research projects, the ongoing COVID-19 pandemic has forced us to reconsider the way we collect data to ensure the safety of our participants and research team. We have developed new approaches to remotely undertake this study. In close cooperation with the ethical review boards evaluating this project, we have developed protocols to ensure that recruitment, consent and rapport building are supported despite the remote nature of data collection. We hope that the procedures outlined in this protocol will spark a discourse on how to conduct ethical and trustworthy research during a pandemic, and that our experiences will allow us to further develop and validate methodological approaches.

FIGURE LEGENDS

Figure 1. Preliminary conceptualization of different influences on vaccination behavior and VH to ground intervention development.

For peer review only - http://bmjopen.bmj.com/site/about/guidelines.xhtml

Figure 2. Location of Cavite, Calabarzon Region included in the Project SALUBONG in the Philippines. Map credits: courtesy of www.mapchart.net

Figure 3. Summary of the Project SALUBONG guided by the human-centered design. Icon credits: all icons made by Freepik (http://www.freepick.com) courtesy of www.flaticon.com

Figure 4. Study design of Phase 4: "Navigating and Gaining". Icon credits: all icons made by Freepik (http://www.freepick.com) courtesy of www.flaticon.com

DECLARATIONS

Acknowledgements: We would like to thank Prof. Dr. Manuela de Allegri and Dr. Veronica Tallo for their expert advice on methodology and local study implementation. Special appreciation is also given to Meghan Obermeyer for her inspiring global health stories, which sparked the idea of this project.

Contributors: MDR participated in all aspects of the study design, set up of local study implementation systems and led the writing of the protocol. JW oversaw the Heidelberg ethics approval process, contributed to and edited the protocol paper. KB participated in the design and writing of the qualitative components and edited the protocol paper. MA, JLG, VE, JL, TAB, MPD, JRG and NDC participated setting up local implementation systems including development of standard operating procedures for online data collection and edited the protocol paper. MT advised on the quantitative study design, sample size calculation, local study implementation and edited the protocol paper. MA advised on the HCD process and edited the protocol paper. RC advised on the quantitative study design, sample size calculation and edited protocol paper. SM supervised all aspects of the study design, the writing and editing of the protocol paper. All authors have read and approved the final version of the manuscript.

Funding: This study has been externally reviewed and funded following a competitive grant application and review process by the Global Grand Challenges, Bill and Melinda Gates Foundation [OPP1217275]. Under the grant conditions of the Foundation, a Creative Commons Attribution 4.0 Generic License has already been assigned to the Author Accepted Manuscript version that might arise from this submission. The funders do not have competing interests and did not play a role in the preparation of this protocol paper.

Competing interests: The authors declare no competing interests. The content is the responsibility of the authors and does not necessarily represent the views of any funder.

Availability of data and materials: Not applicable.

For peer review only - http://bmjopen.bmj.com/site/about/guidelines.xhtml

Patient consent for publication: Not applicable. (This manuscript does not contain any identifiable data.)

Provenance and peer review: Not commissioned; externally peer reviewed

Open access: This is an open access article distributed in accordance with the Creative Commons Attribution Non Commercial (CC BY-NC 4.0) license, which permits others to distribute, remix, adapt, build upon this work non-commercially, and license their derivative works on different terms, provided the original work is properly cited, appropriate credit is given, any changes made indicated, and the use is non-commercial. See: http://creativecommons.org/licenses/by-nc/4.0/.

REFERENCES

1. Omer S, Salmon D, Orenstein W, deHart P, Halsey N. Vaccine Refusal, Mandatory Immunization, and the Risks of Vaccine-Preventable Diseases. N Engl J Med. 2009;360:1981-8.
2. WHO. Immunization coverage. 2020.
3. de Figueiredo A, Simas C, Karafillakis E, Paterson P, Larson HJ. Mapping global trends in vaccine confidence and investigating barriers to vaccine uptake: a large-scale retrospective temporal modelling study. Lancet. 2020.
4. Patel MK, Orenstein WA. Classification of global measles cases in 2013-17 as due to policy or vaccination failure: a retrospective review of global surveillance data. Lancet Glob Health. 2019;7(3):e313-e20.
5. Principi N, Esposito S. Early vaccination: a provisional measure to prevent measles in infants. Lancet Infect Dis. 2019;19(11):1157-8.
6. WHO. European Region loses ground in effort to eliminate measles. . Copenhagen2019.
7. Department of Health. Polio Case Confirmed in the Philippines: DoH To Mount Mass Immunization Campaign Philippines: Department of Health; 2019 [Available from: https://www.doh.gov.ph/node/18012.
8. Group WSW. Report of the SAGE Working Group on Vaccine Hesitancy.; 2014.
9. WHO. Ten threats to global health 2019 2019 [Available from: https://www.who.int/emergencies/ten-threats-to-global-health-in-2019.
10. Betsch C, Schmid P, Heinemeier D, Korn L, Holtmann C, Bohm R. Beyond confidence: Development of a measure assessing the 5C psychological antecedents of vaccination. PLoS One. 2018;13(12):e0208601.
11. Lane S, MacDonald NE, Marti M, Dumolard L. Vaccine hesitancy around the globe: Analysis of three years of WHO/UNICEF Joint Reporting Form data-2015-2017. Vaccine. 2018;36(26):3861-7.
12. Marti M, de Cola M, MacDonald NE, Dumolard L, Duclos P. Assessments of global drivers of vaccine hesitancy in 2014-Looking beyond safety concerns. PLoS One. 2017;12(3):e0172310.
13. Larson H. Stuck: How Vaccine Rumors Start — and Why They Don't Go Away: Oxford University Press; 2020.
14. Dube E, Gagnon D, MacDonald NE, Hesitancy SWGoV. Strategies intended to address vaccine hesitancy: Review of published reviews. Vaccine. 2015;33(34):4191-203.
15. Benecke O, DeYoung SE. Anti-Vaccine Decision-Making and Measles Resurgence in the United States. Glob Pediatr Health. 2019;6:2333794X19862949.
16. WHO. Immunization gaps contribute to rising measles cases in the Philippines Philippines2019 [Available from: https://www.who.int/philippines/news/feature-stories/detail/immunization-gaps-contribute-to-rising-measles-cases-in-the-philippines.
17. The Lancet Infectious D. Infectious disease crisis in the Philippines. Lancet Infect Dis. 2019;19(12):1265.
18. Mello MM, Silverman RD, Omer SB. Ensuring Uptake of Vaccines against SARS-CoV-2. N Engl J Med. 2020;383(14):1296-9.
19. Schaffer DeRoo S, Pudalov NJ, Fu LY. Planning for a COVID-19 Vaccination Program. JAMA. 2020;323(24):2458-9.
20. Sadaf A, Richards JL, Glanz J, Salmon DA, Omer SB. A systematic review of interventions for reducing parental vaccine refusal and vaccine hesitancy. Vaccine. 2013;31(40):4293-304.
21. Jarrett C, Wilson R, O'Leary M, Eckersberger E, Larson HJ, Hesitancy SWGoV. Strategies for addressing vaccine hesitancy - A systematic review. Vaccine. 2015;33(34):4180-90.
22. Brewer N, Chapman G, Rothman A, Leask J, Kempe A. Increasing Vaccination: Putting Psychological Science Into Action. Psychological Science in the Public Interest. 2017;18(3):149-207.
23. Tanne JH. US county bars unvaccinated children from public spaces amid measles emergency. BMJ. 2019;364:l1481.
24. Chamberlain AT, Limaye RJ, O'Leary ST, Frew PM, Brewer SE, Spina CI, et al. Development and acceptability of a video-based vaccine promotion tutorial for obstetric care providers. Vaccine. 2019;37(19):2532-6.
25. Horne Z, Powell D, Hummel JE, Holyoak KJ. Countering antivaccination attitudes. Proc Natl Acad Sci U S A. 2015;112(33):10321-4.
26. Bazzano AN, Martin J, Hicks E, Faughnan M, Murphy L. Human-centred design in global health: A scoping review of applications and contexts. PloS one. 2017;12(11):e0186744.

27. Seeber L, Conrad T, Hoppe C, Obermeier P, Chen X, Karsch K, et al. Educating parents about the vaccination status of their children: A user-centered mobile application. Prev Med Rep. 2017;5:241-50.
28. Henninger ML, McMullen CK, Firemark AJ, Naleway AL, Henrikson NB, Turcotte JA. User-Centered Design for Developing Interventions to Improve Clinician Recommendation of Human Papillomavirus Vaccination. Perm J. 2017;21:16-191.
29. Minoletti S, Rapisarda R, Giraldo L, Grande M, Sommer J, Plazzotta F, et al. User-Centered Design of a Pediatric Vaccination Module for Patients. Stud Health Technol Inform. 2019;264:1096-100.
30. Seeber L, Michl B, Rundblad G, Trusko B, Schnjakin M, Meinel C, et al. A design thinking approach to effective vaccine safety communication. Curr Drug Saf. 2015;10(1):31-40.
31. Inc. JS. VxDel Data Research: Kenya Initial Insights. 2020.
32. Enhancing sustainable access to vaccines for populations in Middle Income Countries (MICs) [Internet]. 2015. Available from: https://www.who.int/immunization/programmes_systems/sustainability/mic_strategy/en/.
33. Muñoz DC, Llamas LM, X. B-C. Exposing concerns about vaccination in low- and middle-income countries: a systematic review. International Journal of Public Health. 2015;60:767-80.
34. Madhav N, Oppenheim B, Gallivan M, Mulembakani P, Rubin E, Wolfe N. Pandemics: Risks, Impacts, and Mitigation. In: rd, Jamison DT, Gelband H, Horton S, Jha P, Laxminarayan R, et al., editors. Disease Control Priorities: Improving Health and Reducing Poverty. Washington (DC)2017.
35. Turner HC, Thwaites GE, Clapham HE. Vaccine-preventable diseases in lower-middle-income countries. Lancet Infect Dis. 2018;18(9):937-9.
36. Larson HJ, Hartigan-Go K, de Figueiredo A. Vaccine confidence plummets in the Philippines following dengue vaccine scare: why it matters to pandemic preparedness. Hum Vaccin Immunother. 2019;15(3):625-7.
37. Fatima K, Syed NI. Dengvaxia controversy: impact on vaccine hesitancy. J Glob Health. 2018;8(2):010312.
38. Dayrit MM, Mendoza RU, Valenzuela SA. The importance of effective risk communication and transparency: lessons from the dengue vaccine controversy in the Philippines. J Public Health Policy. 2020;41(3):252-67.
39. Philippines U-W. Situation Report 3: Measles Outbreak. . Philippines: World Health Organization Philippines; 2019.
40. Migriño J, Gayados B, Birol K, De Jesus L, Lopez C, Mercado W, et al. Factors affecting vaccine hesitancy among families with children 2 years old and younger in two urban communities in Manila, Philippines. Western Pacific Surveillance and Response Journal. 2020;11(2).
41. Dyer O. Philippines measles outbreak is deadliest yet as vaccine scepticism spurs disease comeback. BMJ. 2019;364:l739.
42. IFRC. Operations Update Philippines: Re-emergence of vaccine preventable diseases – Measles outbreak. International Federation of Red Cross and Red Crescent Societies; 2020.
43. DOH identifies Vaccine Hesitancy as one of the reasons for measles outbreak [press release]. Manila, Philippines: Department of Health.
44. UNICEF. Turning doubters into defenders to fight a measles outbreak in the Philippines Philippines2019 [Available from: https://www.unicef.org/philippines/stories/turning-doubters-defenders-fight-measles-outbreak-philippines.
45. Fernandez D. Salubong in Angono, Rizal. Philippine Studies. 1980;28(2):212-20.
46. Alburo J. Boxed In or Out? Balikbayan Boxes as Metaphors for Filipino American (Dis)Location. Ethnologies. 2020;27(2):137-57.
47. Blanc CS. Balikbayan: A Filipino Extension of the National Imaginary and of State Boundaries. Philippine Sociological Review. 1996;44(1):178-93.
48. IDEO.org. The field guide to human-centered design: design kit2015.
49. Fishbein M, Ajzen I. Predicting and Changing Behavior: The Reasoned Action Approach. New York: Psychology Press; 2010.
50. Fahy A, Desmond DM. Irish mothers' intentions to have daughters receive the HPV vaccine. Ir J Med Sci. 2010;179(3):427-30.
51. Malo TL, Hall ME, Brewer NT, Lathren CR, Gilkey MB. Why is announcement training more effective than conversation training for introducing HPV vaccination? A theory-based investigation. Implement Sci. 2018;13(1):57.
52. Britt RK, Hatten KN, Chappuis SO. Perceived behavioral control, intention to get vaccinated, and usage of online information about the human papillomavirus vaccine. Health Psychol Behav Med. 2014;2(1):52-65.
53. Xiao X, Wong RM. Vaccine hesitancy and perceived behavioral control: A meta-analysis. Vaccine. 2020;38(33):5131-8.
54. NIH NIoH. Theory at a Glance: A Guide for Health Promotion Practice (Second Edition). 2005.
55. Kumar S, Quinn SC, Kim KH, Musa D, Hilyard KM, Freimuth VS. The social ecological model as a framework for determinants of 2009 H1N1 influenza vaccine uptake in the United States. Health Educ Behav. 2012;39(2):229-43.
56. Kolff CA, Scott VP, Stockwell MS. The use of technology to promote vaccination: A social ecological model based framework. Hum Vaccin Immunother. 2018;14(7):1636-46.
57. Downs LS, Jr., Scarinci I, Einstein MH, Collins Y, Flowers L. Overcoming the barriers to HPV vaccination in high-risk populations in the US. Gynecol Oncol. 2010;117(3):486-90.
58. Philippines Statistics Office P, ICF. Philippines National Demographic and Health Survey 2017. Quezon City, Philippines, and Rockville, Maryland, USA: PSA and ICF.; 2018.
59. Regional Socio-Deomgraphic Profile [Internet]. [cited October 1, 2020]. Available from: https://rpo4a.popcom.gov.ph/2015-05-26-08-42-52/2015-05-26-09-21-46?showall=1.
60. Services O. Philippines: Measles Outbreak - Feb 2019 2019 [Available from: https://reliefweb.int/disaster/ep-2019-000112-phl.
61. News O. Measles: Calabarzon reports 3,835 percent increase in 2019. Outbreak News Today. 2019.
62. Group OB. Calabarzon a key industrial region, adding significantly to the Philippines' GDP [Available from: https://oxfordbusinessgroup.com/analysis/surpassing-expectations-calabarzon-key-industrial-growth-region-contributing-significantly-national.
63. EMB EMB. CALABARZON [Available from: http://calabarzon.emb.gov.ph/about-us/.
64. Yamashita T, Suplido SA, Llave C, Tuliao MT, Tanaka Y, Matsuo H. Understanding Postpartum Healthcare Services and Exploring the Challenges and Motivations of Maternal Health Service Providers in the Philippines: a Qualitative Study. Trop Med Health. 2015;43(2):123-30.
65. Creswell J. Research Design: Qualitative, Quantitative, and Mixed Methods Approaches. 4th ed. United States of America: Sage Publications; 2014.
66. Dicicco-Bloom B, Crabtree BF. The qualitative research interview. Med Educ. 2006;40(4):314-21.
67. Vasileiou K, Barnett J, Thorpe S, Young T. Characterising and justifying sample size sufficiency in interview-based studies: systematic analysis of qualitative health research over a 15-year period. BMC Med Res Methodol. 2018;18(1):148.
68. Larson HJ, Jarrett C, Schulz WS, Chaudhuri M, Zhou Y, Dube E, et al. Measuring vaccine hesitancy: The development of a survey tool. Vaccine. 2015;33(34):4165-75.

For peer review only - http://bmjopen.bmj.com/site/about/guidelines.xhtml

69. Lin FH, Tsai SB, Lee YC, Hsiao CF, Zhou J, Wang J, et al. Empirical research on Kano's model and customer satisfaction. PLoS One. 2017;12(9):e0183888.
70. Agency EM. Guidance on the management of clinical trials during the COVID-19 (Coronavirus) Pandemic 2020.
71. Force I-AT. Inter-Agency Task Force for the management of emerging infectious diseases: Resolution No. 69-A. Manila, Philippines: Official Gazette; 2020.
72. McMahon SA, Winch PJ. Systematic debriefing after qualitative encounters: an essential analysis step in applied qualitative research. BMJ Glob Health. 2018;3(5):e000837.
73. Charmaz K. Constructing Grounded Theory: A practical guide through qualitative analysis. David S, editor. London, Great Britain: SAGE Publications Ltd; 2006.
74. Greenberg J, Dube E, Driedger M. Vaccine Hesitancy: In Search of the Risk Communication Comfort Zone. PLoS Curr. 2017;9.
75. Jennings FJ, Russell FM. Civility, credibility, and health information: The impact of uncivil comments and source credibility on attitudes about vaccines. Public Underst Sci. 2019;28(4):417-32.
76. Freed GL, Clark SJ, Butchart AT, Singer DC, Davis MM. Sources and perceived credibility of vaccine-safety information for parents. Pediatrics. 2011;127 Suppl 1:S107-12.
77. Carrieri V, Madio L, Principe F. Vaccine hesitancy and (fake) news: Quasi-experimental evidence from Italy. Health Econ. 2019;28(11):1377-82.
78. Burki T. Vaccine misinformation and social media. The Lancet Digital Health. 2019;1(6):E258-E9.
79. Steffens MS, Dunn AG, Wiley KE, Leask J. How organisations promoting vaccination respond to misinformation on social media: a qualitative investigation. BMC Public Health. 2019;19(1):1348.
80. Daley MF, Narwaney KJ, Shoup JA, Wagner NM, Glanz JM. Addressing Parents' Vaccine Concerns: A Randomized Trial of a Social Media Intervention. Am J Prev Med. 2018;55(1):44-54.
81. Glanz JM, Wagner NM, Narwaney KJ, Kraus CR, Shoup JA, Xu S, et al. Web-based Social Media Intervention to Increase Vaccine Acceptance: A Randomized Controlled Trial. Pediatrics. 2017;140(6).
82. Penta MA, Baban A. Message Framing in Vaccine Communication: A Systematic Review of Published Literature. Health Commun. 2018;33(3):299-314.
83. Hendrix KS, Finnell SM, Zimet GD, Sturm LA, Lane KA, Downs SM. Vaccine message framing and parents' intent to immunize their infants for MMR. Pediatrics. 2014;134(3):e675-83.

For peer review only

For peer review only - http://bmjopen.bmj.com/site/about/guidelines.xhtml

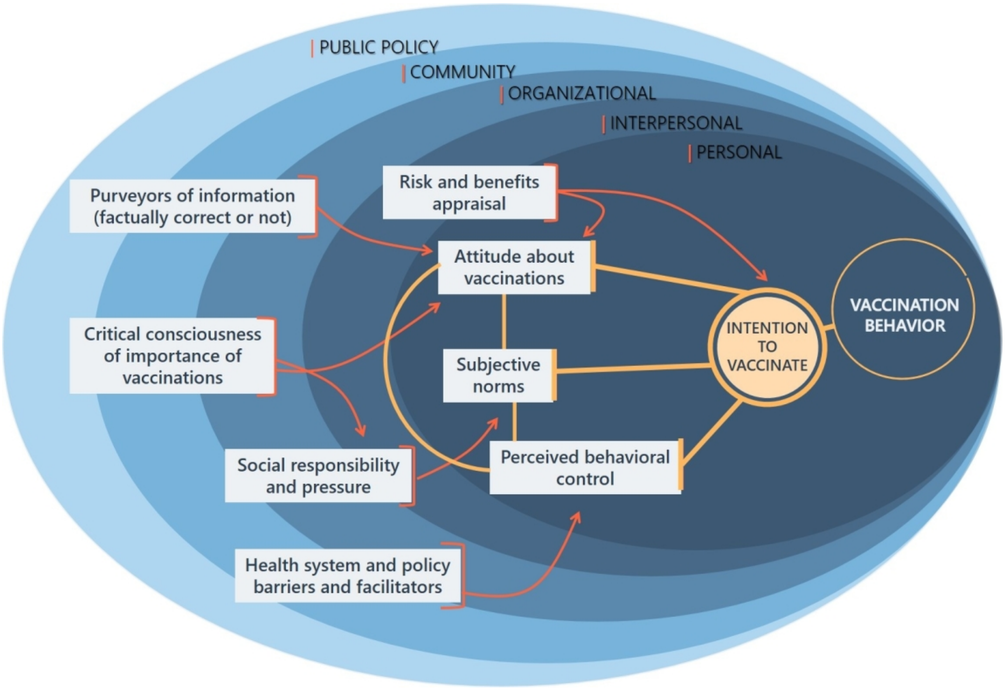

Figure 1. Preliminary conceptualization of different influences on vaccination behavior and VH to ground intervention development.

272x186mm (150 x 150 DPI)

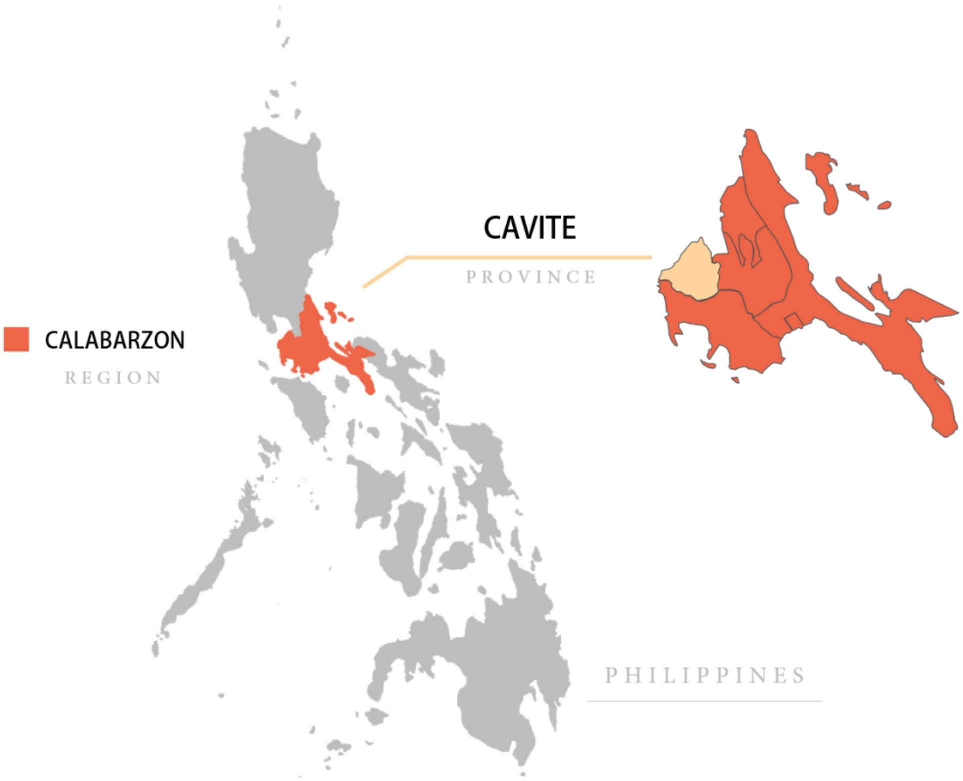

Figure 2. Location of Cavite, Calabarzon Region included in the Project SALUBONG in the Philippines. Map credits: courtesy of www.mapchart.net

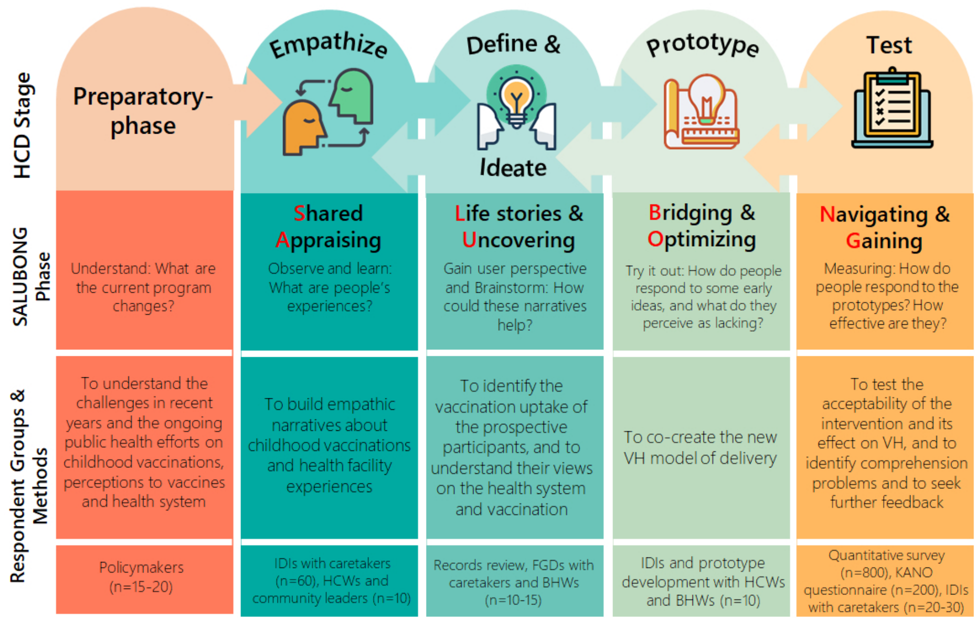

Figure 3. Summary of the Project SALUBONG guided by the human-centered design. Icon credits: all icons made by Freepik (http://www.freepik.com) courtesy of www.flaticon.com

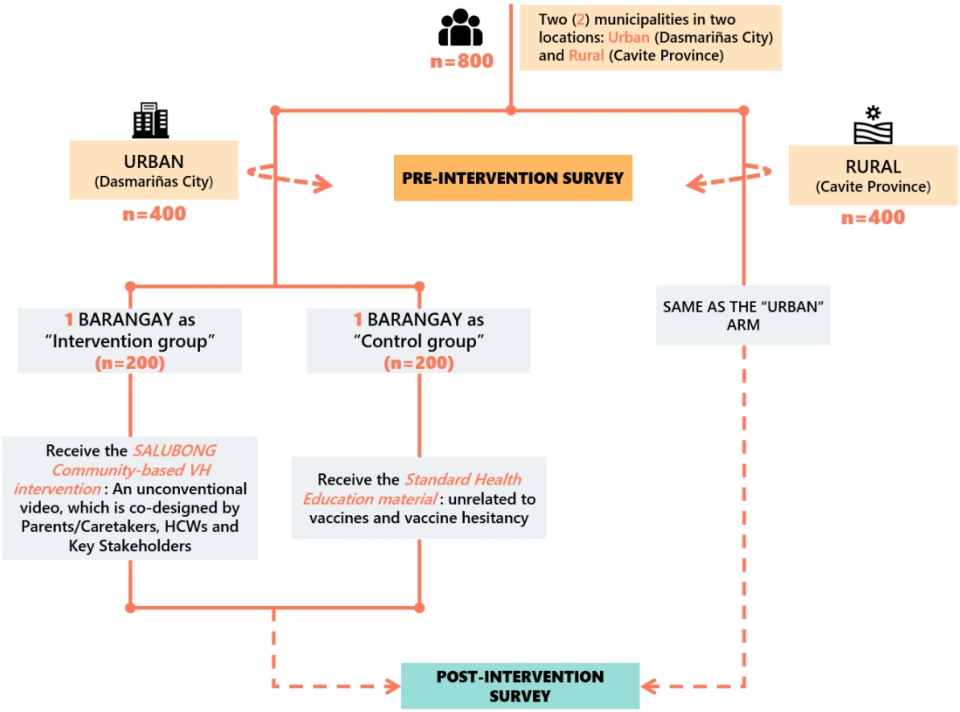

Figure 4. Study Design of Phase 4: "Navigating and Gaining". Icon credits: all icons made by Freepik (http://www.freepik.com) courtesy of www.flaticon.com.
